# Synthesis of *p*‐Coumarates With Potential Anti‐Alzheimer's Action: Enzyme Inhibition and In Silico Studies

**DOI:** 10.1002/cbdv.202503857

**Published:** 2026-04-01

**Authors:** Susiany Pereira Lopes, Jeremias Justo Emídio, Allana Brunna Sucupira Duarte, Ilkay Erdogan Orhan, Fatma Sezer Senol Deniz, Ramin Ekhteiari Salmas, Damião Pergentino de Sousa

**Affiliations:** ^1^ Department of Pharmaceutical Sciences Federal University of Paraíba João Pessoa Paraíba Brazil; ^2^ Faculty of Pharmacy Department of Pharmacognosy Lokman Hekim University Ankara Türkiye; ^3^ Inovabella Biotechnology and R&D Industry and Trade Corp., LHUSTEK Ankara Türkiye; ^4^ Faculty of Pharmacy Department of Pharmacognosy Gazi University Ankara Türkiye; ^5^ Department of Chemistry Britannia House, King's College London UK

**Keywords:** acetylcholinesterase, Alzheimer's disease, butyrylcholinesterase, cholinesterase inhibitors, natural product

## Abstract

Alzheimer's disease (AD) is a fatal neurodegenerative disorder that affects cognition, memory, and behavior. Such a disease is considered the most common cause of dementia and affects a large portion of the elderly population worldwide. Currently, cholinesterase inhibitors are used to reduce the symptoms and rate of progression of this disease. Thus, the present study evaluated the acetylcholinesterase (AChE) and butyrylcholinesterase (BChE) inhibitory activities of a set of 22 *p*‐coumarate derivatives using the spectrophotometric method. The inhibitory activity of the compounds against AChE and BChE was measured using the adapted Ellman spectrophotometric method; the reported inhibition percentages were determined at a final concentration of 100 µM. The structures of the synthesized compounds were characterized by FTIR, ^1^H‐NMR, ^13^C‐NMR, and HRMS spectroscopy. Among the compounds tested, three showed moderate inhibitory activity against AChE and good activity against BChE: (*E*)‐4‐chlorobenzyl 3‐(4‐hydroxyphenyl)acrylate (**14**) (56.36%; 75.17%), (*E*)‐4‐bromobenzyl 3‐(4‐hydroxyphenyl)acrylate (**15**) (61.11%; 76.09%), and (*E*)‐naphthalene 3‐(4‐hydroxyphenyl)acrylate (**18**) (59.18%; 65.39%), respectively. Compound **15** had an IC_50_ of 22.22 ±1.50 mM against BChE, which is notably better than galantamine's BChE inhibition. The in silico analysis suggested that compounds **14**, **15**, and **18** interact with AChE and BChE. Thus, *p*‐coumaric acid derivatives represent promising prototypes for the search for new drug candidates for the treatment of AD.

## Graphical abstract

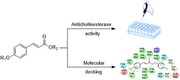

## 1 Introduction

Alzheimer's disease (AD) is a degenerative brain disease that affects the memory, cognitive skills, and everyday activities, leading to dementia [1, 2]. Due to the increase in the elderly population, the number of AD patients could reach approximately 106.8 million people worldwide by 2050 [3]. In the pathophysiology of the disease, there is the destruction of neuronal destruction, resulting in lower performance of the brain [4, 5, 6]. Among the various factors contributing to this pathology are decreased acetylcholine levels due to cholinergic deficit [7, 8]. Although the etiology of the disease is not completely understood, the most common pharmacotherapy involves cholinesterase inhibitors (ChEIs) (rivastigmine, tacrine, galantamine, and donezepil), which block acetylcholinesterase (AChE) and butyrylcholinesterase (BChE), enzymes responsible for acetylcholine hydrolysis. By increasing acetylcholine levels, these inhibitors help improve disease symptoms, but their use is limited due to side effects [9, 10].

ChEIs represent a cornerstone in the symptomatic management of AD. Their therapeutic value lies in their ability to ameliorate the cholinergic neurotransmitter deficit, a well‐established feature of AD and related dementias. These agents reversibly inhibit AChE, the enzyme responsible for acetylcholine hydrolysis in the synaptic cleft. By preventing acetylcholine breakdown, ChEIs increase the concentration and duration of action of this crucial neurotransmitter at central cholinergic synapses. Nevertheless, they may cause side effects resulting from the stimulation of muscarinic and nicotinic receptors in the autonomic nervous system, neuromuscular junctions, and brain. Due to the side effects (i.e., nausea, vomiting, anorexia, dyspepsia, muscle cramps, fatigue, headache, and dizziness) of currently available drugs, the search for new and more effective ChEIs remains a priority for AD treatment [11].

Cinnamic acid derivatives are naturally occurring compounds with diverse biological properties relevant to neurological disorders [12]. Among these derivatives, *p*‐coumaric acid (hydroxycinnamic acid) is a phenolic compound widely used in the cosmetic, food, chemical, and pharmaceutical industries. It exhibits numerous biological activities, including antioxidant, anti‐inflammatory, antiplatelet, antimicrobial, antidiabetic, and potential anti‐Alzheimer's effects [13, 14, 15]. Previous studies developed by Nugroho et al., Ozer et al., and Thuphairo et al. demonstrated that *p*‐coumaric acid isolated from plant extracts displays anticholinesterase activity [16, 17, 18]. Therefore, a series of *p*‐coumaric acid derivatives was prepared and evaluated against AChE and BChE enzymes to explore the anti‐Alzheimer potential of this chemical class. An in silico approach was employed to simulate possible interactions with the enzymes.

## 2 Results and Discussion

### 2.1 Chemistry of Compounds **1–22**

**SCHEME 1:**
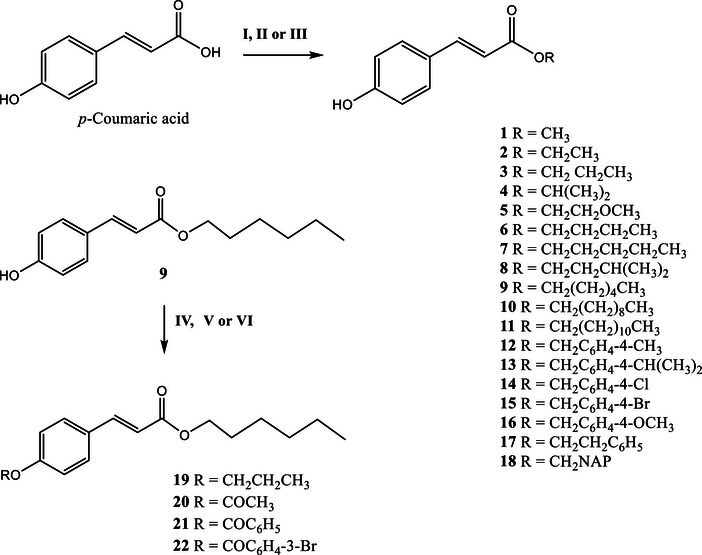
Preparation of the *p*‐coumaric acid derivatives: (**I**) ROH, H_2_SO_4_, reflux; (**II**) ROH, THF, TPP, DIAD, 0°C to r.t.; (**III**) Et_3_N, halide, acetone, reflux; (**IV**) BrCH_2_CH_2_CH_3_, K_2_CO_3_, acetone, reflux; (**V**) pyridine, acetic anhydride, r.t.; (**VI**) acid halide, NaOH, r.t.

Compounds **1**–**8** were prepared by Fischer esterification, and compounds **9** and **11**–**13** were synthesized via the Mitsunobu reaction. The compounds were characterized by infrared (IR) and nuclear magnetic resonance (NMR) spectroscopy, as previously reported by Lopes et al. [19]. Compounds **10** and **14**–**18** were obtained through halide reactions, while compounds **19**–**22** were synthesized via reactions involving ethers and esters of hexyl *p*‐coumarate (Scheme 1).

### 2.2 Anticholinesterase Activity of Compounds **1–22**

The results are presented as percentages of inhibition and standard deviation (S.D.), along with the concentration required to inhibit 50% of the enzyme activity (IC_50_). A set of 22 compounds were analyzed for their inhibitory activity against AChE and BChE. As shown in Table 1, three compounds (**14**, **15**, and **18**) exhibited more potent inhibitory activity against BChE, while the others displayed weak to moderate activity against both ChEs.

**TABLE 1:** AChE and BChE inhibitory activity and molar mass of compounds **1–22**.

	Inhibition % ± SD^a^ at 100 µM^b^		Molar mass (g/mol)	Selectivity index for AChE
	AChE	BChE		
**1**	—^c^	3.39 ± 0.25	178.18	
**2**	—	—	192.21	
**3**	—	2.63 ± 0.26	206.23	
**4**	—	11.14 ± 0.02	206.23	
**5**	—	2.65 ± 0.50	222.23	
**6**	31.07 ± 2.75	31.92 ± 2.92	220.26	
**7**	8.43 ± 1.71	19.74 ± 1.76	234.29	
**8**	8.04 ± 2.70	27.48 ± 1.19	234.29	
**9**	24.02 ± 2.07	27.92 ± 1.26	248.31	
**10**	28.30 ± 0.99	16.02 ± 1.50	304.43	
**11**	17.59 ± 1.32	—	332.47	
**12**	29.04 ± 1.72 (IC_50_ > 100 µM)	55.02 ± 0.60 (IC_50_ = 85.61 ± 3.18 µM)	268.30	< 0.8561
**13**	20.74 ± 2.15	34.33 ± 2.80	296.36	
**14**	56.36 ± 2.27 (IC_50_ = 87.31 ± 2.76 µM)	75.17 ± 1.81 (IC_50_ = 19.08 ± 0.70 µM)	288.72	0.218
**15**	61.11 ± 1.13 (IC_50_ = 72.64 ± 1.30 µM)	76.09 ± 2.16 (IC_50_ = 22.22 ± 1.50 µM)	333.17	0.305
**16**	36.44 ± 1.06 (IC_50_ > 100 µM)	50.31 ± 2.12 (IC_50_ = 88.52 ± 2.72 µM)	284.30	< 0.8852
**17**	41.30 ± 2.15 (IC_50_ > 100 µM)	50.24 ± 2.06 (IC_50_ = 103.25 ± 2.47 µM)	268.30	< 1.0325
**18**	59.18 ± 2.82 (IC_50_ = 54.72 ± 2.78 µM)	65.39 ± 3.88 (IC_50_ = 22.69 ± 2.15 µM)	304.33	0.414
**19**	3.18 ± 1.93	9.15 ± 1.23	290.39	
**20**	3.57 ± 0.63	16.08 ± 2.36	290.35	
**21**	—	3.07 ± 0.96	352.42	
**22**	7.44 ± 1.05	—	431.31	
**Galantamine hydrobromide (reference)**	91.03 ± 1.36 (IC_50_ = 4.04 ± 0.16 µM)	49.01 ± 2.01 (IC_50_ = 86.75 ± 1.59 µM)		

^a^ Standard deviation (*n* = 4).

^b^ Final concentration.

^c^ No inhibition.

In AChE, the peripheral anionic site (PAS) is defined by a dense network of π–π stacking interactions with Tyr72, Trp286, and Tyr341, while a hydrogen bond with Asp74 provides an additional anchor at the gorge entrance. Deeper within the enzyme, the catalytic anionic site (CAS) is engaged in π–π stacking with Phe338, a hydrogen bond with the Phe295 backbone, and direct interaction with the catalytic residue His447. A similar dual‐site engagement occurs in BChE, though involving fewer residues; the PAS is secured via a hydrogen bond with Asp70, while the CAS is anchored by π–π stacking with Trp231 and Phe329, as well as a critical hydrogen bond with the catalytic His438.

Based on the analysis of the results, a clear correlation can be observed between the experimental anticholinesterase activities and the molecular docking outcomes. Compounds **14**, **15**, and **18**, which exhibited the highest inhibition percentages and the lowest IC_50_ values—particularly against BChE—also displayed the most favorable binding energies in induced‐fit docking simulations. These compounds established hydrogen bonds as well as extensive hydrophobic and π–π interactions with key residues in the active site and peripheral regions of AChE and BChE. The stronger predicted binding affinities toward BChE are consistent with the superior experimental inhibitory profiles, thereby corroborating the structure–activity relationship and validating the docking results as a rational explanation for the observed biological activity.

The introduction of alkyl moieties into the *p*‐coumaric acid scaffold was guided by structure–activity relationship (SAR) considerations mainly aimed at modulating physicochemical properties and steric effects, rather than by an a priori assumption of enhanced acetylcholinesterase (AChE) or butyrylcholinesterase (BChE) inhibition [20]. In agreement with the results of the present study, which show that simple alkyl esters generally exhibit weak to moderate inhibitory activity without a clear correlation between alkyl chain length and potency, previous investigations evaluating phenolic acids and their alkyl esters have reported limited and non‐linear effects on AChE and BChE activity, emphasizing that the phenolic core and its ability to establish specific interactions with the enzyme are more determinant than alkyl chain length [20]. Moreover, investigations on cinnamic acid and *p*‐coumarate derivatives indicate that substituents capable of establishing aromatic and π–π interactions within the enzyme active site contribute more effectively to cholinesterase inhibition than simple alkyl groups [3]. Therefore, in the present work, alkyl moieties were explored primarily as modulators of the *p*‐coumaric pharmacophore, whereas the inhibitory activity observed for bioactive derivatives is more consistently associated with aromatic substituents than with alkyl chain elongation [3].

In the present study, compounds bearing short linear alkyl chains exhibited either weak or no inhibitory activity against AChE and BChE. Compound **1**, (*E*)‐methyl 3‐(4‐hydroxyphenyl)acrylate, which contains a methyl group in the side chain, showed no inhibition of AChE and only weak inhibition of BChE (3.39% ± 0.25%). Compound **2**, (*E*)‐ethyl 3‐(4‐hydroxyphenyl)acrylate, with an ethyl side chain, did not exhibit any anticholinesterase activity. In contrast, methyl cinnamate—a structurally related compound lacking a hydroxyl group at the para position of the aromatic ring—has been reported to inhibit ChE activity. Similarly, ethyl ferulate, which bears a hydroxyl group at the para position and a methoxy group at the meta position, demonstrated a moderate inhibitory activity against both AChE and BChE in previous studies [21, 22]. Compound **3**, which contains a propyl group as a side chain, demonstrated a weak inhibition against BChE (2.63% ± 0.26%). Additionally, Zhao et al. reported that propyl gallate, bearing hydroxyl groups at the ortho and para positions of the aromatic ring, exhibited inhibitory activity against AChE [23].

Although compounds **3** and **4**, which contain propyl and isopropyl substituent groups, respectively, showed different inhibitory potencies against BChE. A comparison of their chemical structures suggests that the insertion of a branched alkyl group was responsible for the increased inhibitory activity against BChE [24]. Thus, compound **4** inhibited BChE activity by 11.14% ± 0.02%, while compound **3** inhibited only 2.63% ± 0.26%. Compound **5**, which has a 2‐methoxyethyl substituent—and therefore includes a heteroatom in the ester side chain—inhibited 2.65% ± 0.50% of BChE enzymatic activity, displaying reduced anticholinesterase activity compared to that of compound **4**. Among the alkyl esters, compound **6** [(*E*)‐butyl 3‐(4‐hydroxyphenyl)acrylate], which contains a butyl side chain, inhibited both enzymes and showed higher activity against BChE, with inhibition values of 31.07% and 31.92% against AChE and BChE, respectively. This effect may be related to the high lipophilicity of this compound [25].

Comparing the enzymatic activity data of compound **6** to that of compound **7**, the latter bearing a pentyl side chain and thus possessing a longer and more lipophilic alkyl chain, it was observed that this structural modification led to decreased anticholinesterase activity with inhibition values of 8.43% ± 1.71% and 19.74% ± 1.76% against AChE and BChE, respectively. Compound **8**, featuring an isopentyl side chain, exhibited weak inhibitory activity toward AChE (8.04% ± 2.70% inhibition) but demonstrated moderate inhibition of BChE (27.48% ± 1.19%). As exemplified by compound **8**, this modification has a limited impact on AChE inhibition, whereas a more pronounced effect is observed on BChE activity.

**FIGURE 1:**
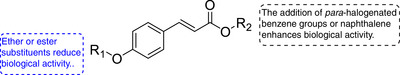
The structure‐activity relationship of p‐coumarates and their biological activity.

Compound **9**, that is, hexyl *p*‐coumarate, which contains a linear six‐carbon hexyl side chain, exhibited inhibitory activity of 24.02% ± 2.07% against AChE and 27.92% ± 1.26% against BChE. However, its inhibitory effect was comparable to that of compound **8**, which features a pentyl substituent. Compound **10**, bearing a decyl side chain, inhibited 28.30% ± 0.99% of AChE activity, suggesting a modest increase in biological potency against this enzyme with increased lipophilicity; in contrast, it inhibited only 16.02% ± 1.50% of BChE activity, representing a decrease compared to compound **9**. Compound **11**, with an even longer unbranched dodecyl chain, inhibited 17.59% ± 1.32% of AChE activity, indicating that excessive lipophilicity may compromise inhibitory capacity. Interestingly, despite the reduced activity observed for highly lipophilic derivatives in the present study, a structurally related cinnamic acid derivative—[12‐(4‐hydroxy‐3‐methyl‐oxo‐cyclopenta‐1,3‐dien‐1‐yl)‐11‐methyl‐dodecyl] (*E*)‐3‐(3,4‐dimethylphenyl)prop‐2‐enoate—was reported by Elufioye et al. to exhibit anticholinesterase activity [26] (Figure 1).

Enzyme inhibition assays demonstrated that compounds containing benzyl substituents had weak to moderate inhibitory activity against AChE and moderate to marked activity against BChE. The insertion of benzyl substituents in the side chain enhanced the effectiveness of the compounds. This is evident in the enzyme inhibitory profiles of compounds **12–18**, in which anticholinesterase activity was favored by the presence of such substituents. Compound **12**, with a 4‐methylbenzyl side chain, inhibited 29.04 ± 1.72% of AChE activity and 55.02% ± 0.60% of BChE activity. These results indicate that the introduction of benzyl groups enhances the biological activity compared to alkyl substituents. However, compound **13**, which contains a 4‐isopropylbenzyl group, exhibited reduced anticholinergic activity, inhibiting 20.74% ± 2.15% and 34.33% ± 2.80% of AChE and BChE activity, respectively, values considerably lower than those observed for compound **12**. Therefore, the insertion of a branched alkyl chain, such as isopropyl, at the para position of the benzyl substituent results in reduced activity. Introducing benzyl substituents significantly enhances activity compared to simple alkyl substituents.

Upon analyzing the biological activity of compound **14**, which features a 4‐chlorobenzyl group in its side chain, it becomes apparent that the presence of the chlorine atom contributed to the anticholinesterase activity. This compound inhibited 56.36% ± 2.27% and 75.17% ± 1.81% of AChE and BChE activity, respectively, demonstrating greater potency than compound **12**. Compound **15**, with a 4‐bromobenzyl group, inhibited 61.11% ± 1.13% and 76.09% ± 2.16% of AChE and BChE activity, respectively. This activity was very similar to that observed for compound **14** against BChE, though slightly higher against AChE. These results are consistent with previous studies, which found that substitution with electronegative groups such as chlorine and bromine on the aromatic ring favored inhibitory activity against BChE [27, 28].

**FIGURE 2:**
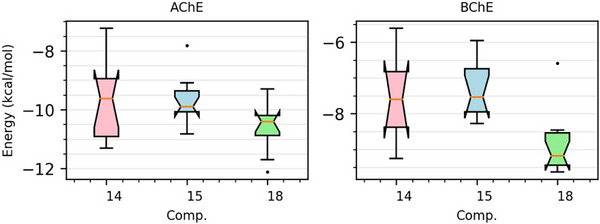
Induced fit docking scores of 4‐chlorobenzyl *p*‐coumarate (**14**), 4‐bromobenzyl *p*‐coumarate (**15**), and naphthalene *p*‐coumarate (**18**).

**FIGURE 3:**
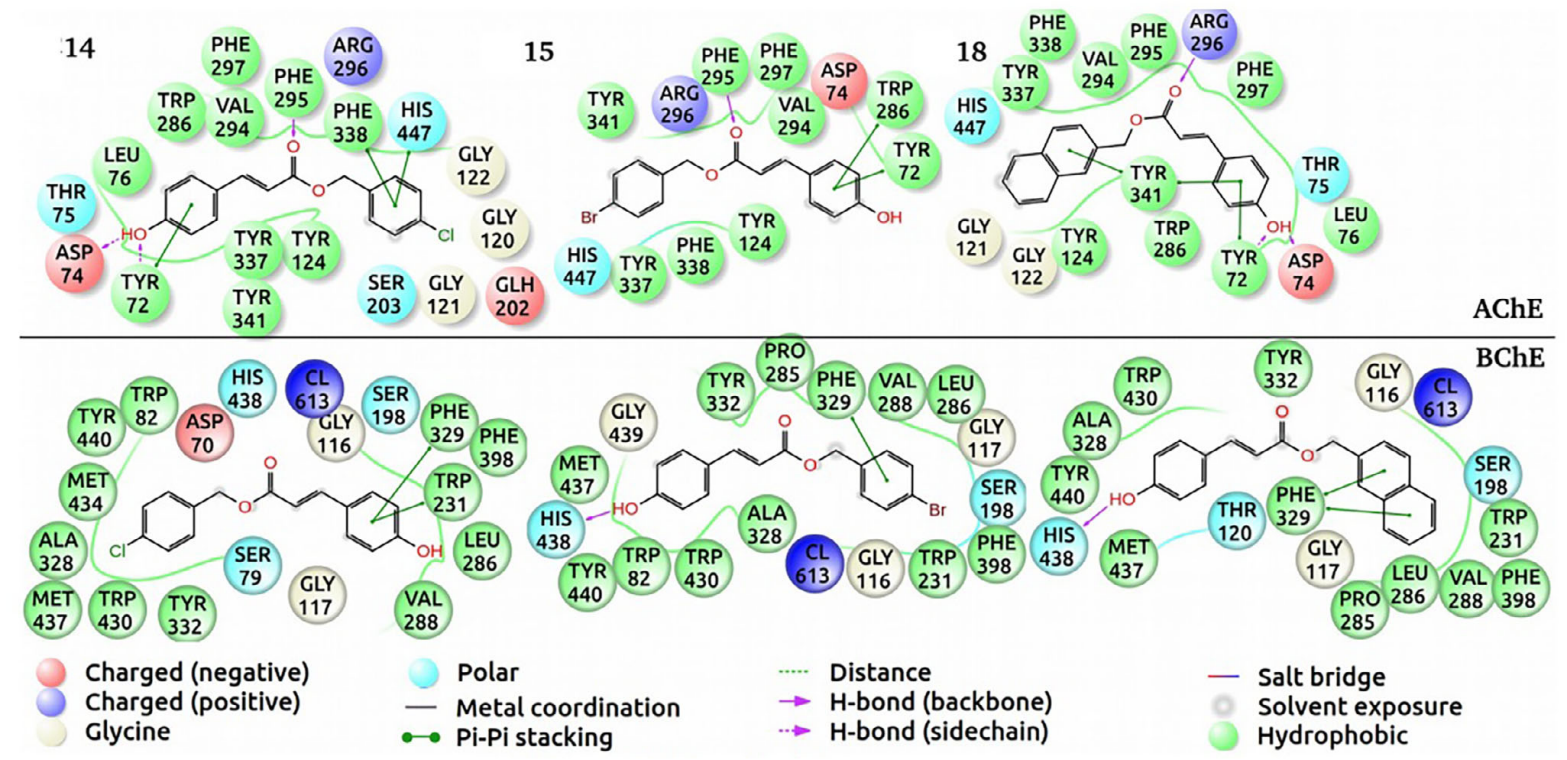
Top docking score poses of 4‐chlorobenzyl *p*‐coumarate (**14**), 4‐bromobenzyl *p*‐coumarate (**15**), and naphthalene *p*‐coumarate (**18**).

The in silico docking studies were conducted to elucidate the binding modes and affinities of the most active compounds (**14**, **15**, and **18**) against AChE (PDB: 4EY7) and BChE (PDB: 5DYW). The induced fit docking (IFD) approach was employed to account for ligand and receptor flexibility, providing more reliable binding poses and energy estimates. The calculated ligand‐binding energies for compounds **14**, **15**, and **18** against AChE were –9.2, –9.5, and –9.8 kcal/mol, respectively, while against BChE, they were –10.1, –10.3, and –10.6 kcal/mol, respectively. While the docking scores do not strictly mirror the IC50 trends, the predicted binding orientations remain highly favorable and consistent with the potent inhibition observed for AChE and BChE. Molecular docking results (Figures 2 and 3), the hydroxyl group of compound **14** forms hydrogen bonds with amino acids ASP 74 and TYR 72, in addition to hydrophobic interactions with LEU 76, TRP 286, VAL 294, PHE 297, PHE 295, PHE 338, TYR 341, TYR 337, and TYR 124, thereby inhibiting AChE. Regarding BChE inhibition, hydrophobic interactions occurred with amino acids TRP 82, TYR 440, MET 434, ALA 328, MET 437, TRP 430, TYR 332, PHE 329, PHE 398, TRP 231, LEU 286, and VAL 288. Compound **15**, in turn, exhibited hydrophobic interactions with TYR 341, PHE 295, PHE 297, VAL 294, TRP 286, TYR 72, TYR 124, PHE 338, and TYR 337, thereby inhibiting AChE. For BChE inhibition, several hydrophobic interactions were formed with TYR 332, PRO 285, PHE 329, VAL 288, LEU 286, PHE 398, TRP 231, ALA 328, TRP 430, TRP 82, and TYR 440, in addition to a hydrogen bond between the hydroxyl group of compound **15** and HIS 438.

Compound **16**, bearing a 4‐methoxybenzyl group, exhibited reduced inhibitory activity against AChE and BChE, with inhibition rates of 36.44% ± 1.06% and 50.31% ± 2.12%, respectively. Compared to analogues containing electron‐withdrawing substituents at the *para* position of the benzyl moiety, compound **16** demonstrated lower biological potency, likely due to the electron‐donating nature of the methoxy group. Compound **17**, which incorporates a phenethyl group at the same position, exhibited a similar inhibitory profile against BChE (50.24% ± 2.06%), but enhanced activity against AChE (41.30% ± 2.15%). The introduction of a naphthyl group in compound **18** significantly increased inhibitory activity, with inhibition rates of 59.18% ± 2.82% (AChE) and 65.39% ± 3.88% (BChE), highlighting the contribution of the aromatic system to enhanced binding affinity, possibly due to increased molecular volume, lipophilicity, and π–π stacking interactions. Compound **18** also displayed a superior inhibitory profile (IC_50_ = 22.69 µM) compared to the reference drug galantamine (IC_50_ = 86.75 µM). These findings align with previous studies; Takao et al. reported BChE inhibition by phenethyl *p*‐coumarate, while Gießel et al. and Gülçin et al. observed dual ChE inhibition by phenethyl caffeate [29, 30]. Molecular docking studies further supported the experimental data, revealing that the hydroxyl group of compound **18** forms hydrogen bonds with AChE residues, for example, ASP 74 and TYR 72, along with hydrophobic interactions involving TYR 337, PHE 338, VAL 294, PHE 295, PHE 297, TYR 341, TRP 286, and TYR 124. In BChE, similar hydrophobic interactions were observed with TYR 440, ALA 328, TRP 430, TYR 332, TRP 231, PHE 398, VAL 288, LEU 286, PRO 285, PHE 329, and MET 437, in addition to a hydrogen bond between the hydroxyl group and HIS 438.

Compounds **19–22** feature a hexyl group as a side chain; therefore, their structural differences lie in the substitution of the hydrogen atom of the hydroxyl group at the *para* position of the aromatic ring with alkyl or aromatic substituents. The results suggest that the hexyl group in the side chain contributed minimally to the biological activity and, in fact, reduced the potency of the compounds, likely due to its bulky nature and high lipophilicity. Additionally, the various substituents at the *para* position had a limited influence on activity. Therefore, the replacement of the hydroxyl group in hexyl *p*‐coumarate with aliphatic or aromatic groups negatively affected anticholinesterase activity [25, 31, 32, 33, 34].

Further increasing the chain length (compounds **7**, **9–11**) generally led to reduced activity against BChE, while inconsistent activity against AChE suggests an optimal chain length for *p*‐coumarate derivatives, likely due to geometric constraints in the binding pocket or excessive lipophilicity compromising solubility and delivery.

## 3 Conclusions

Among the series of *p*‐coumarates studied, compounds **14**, **15**, and **18** stood out due to their remarkable inhibitory potency against AChE and BChE. Compound **14** inhibited 56.36% ± 2.27% and 75.17% ± 1.81% of AChE and BChE enzymatic activity, respectively. Compound **15** inhibited 61.11% ± 1.13% of AChE activity and 76.09% ± 2.16% of BChE activity. Finally, compound **18** inhibited 59.18% ± 2.82% and 65.39% ± 3.88% of AChE and BChE activity, respectively. Structure‐activity relationship analyses revealed that the presence of a *para*‐substituted phenyl ring contributes to the bioactivity of the compounds. Our results strongly support that compounds **14**, **15**, and **18** are promising prototypes for AD drug development. Therefore, further studies are needed to evaluate the potential of *p*‐coumaric acid derivatives in the development of new drugs for the treatment of AD.

## 4 Materials and Methods

### 4.1 Reagents and Chemical Characterization

The derivatives were purified by column adsorption chromatography (CC) using silica gel 60, ART 7734 – MERCK, St. Louis, Missouri, EUA. Infrared spectra were performed using FTIR spectrophotometry, and ^1^H and ^13^C NMR spectra were obtained using a Bruker Ascend spectrometer (Bruker, Bremen, Germany), operating at 400 and 100 MHz, respectively.

### 4.2 General Procedure for Preparation of Compounds **1–8**

*p*‐Coumaric acid (0.1 g; 0.61 mmol) in alcohol (20 mL) in the presence of H_2_SO_4_ (0.2 mL) was refluxed (5–27 h), as published by Lopes et al. [19, 35].

### 4.3 Preparation of Compounds **9, 11–13** by Mitsunobu Reaction

*p*‐Coumaric acid (0.1 g; 0.61 mmol) and alcohol (0.61 mmol) in 2.25 mL of tetrahydrofuran were stirred under magnetic stirring at 0°C for 30 min. Diisopropyl azodicarboxylate (0.12 mL; 0.61 mmol) and triphenylphosphine (0.16 g; 0.61 mmol) were added. The mixture was stirred at room temperature for 48–52 h and monitored by TLC [19, 35, 36].

### 4.4 Preparation of Compounds **10, 14–18**

*p*‐Coumaric acid (0.4 g; 2.43 mmol) in acetone (29.5 mL) was heated under reflux in the presence of triethylamine (1.31 mL) and halide (2.51 mmol) until complete reaction (48 h). The solvent was evaporated under reduced pressure, and 20 mL of water was added to the solution. The solution was then extracted with dichloromethane (3 × 20 mL) and washed with water (20 mL). The organic phase was dried over anhydrous sodium sulfate, and the solvent was then evaporated under reduced pressure. The product was isolated by CC on a silica gel 60 using hexane and ethyl acetate (8:2) as eluents [36]. Compounds **10, 14, 16,** and **17** have been previously described [36, 37, 38, 39].

*(E*)‐4‐Bromobenzyl *p*‐coumarate (**15**): Amorphous white solid; yield: 15.71% (127.6 mg; 0.38 mmol); mp: 122°C–123°C; *R*_f_ = 0.62 (hexane/EtOAc [8:2]); IR (KBr, *ν*_max_, cm^−^^1^): 3372, 3023, 2952, 1686, 1625, 1605, 1459, 1274, 1170, and 1046; ^1^H NMR (500 MHz, CDCl_3_): δH 7.66 (*d*, *J* = 15.00 Hz, 1H), 7.50 (*d*, *J* = 10.00 Hz, 2H), 7.41 (*d*, *J* = 10.00 Hz, 2H), 7.28 (*d, J* = 10.00 Hz, 2H), 6.84 (*d*, *J* = 10.00 Hz, 2H), 6.32 (*d, J* = 15.00 Hz, 1H), 5.19 (*s*, 2H); ^1^ ^3^C NMR (100 MHz, CDCl_3_): δC 167.5, 158.3, 145.6, 135.3, 131.9, 130.2, 130.0, 127.0, 122.4, 116.1, 115.0, 65.8; HRMS (MALDI) calculated for C_16_H_13_BrO_3_ [M + H]^+^: 333.0126; found: 333.0120.

(*E*)‐Naphthalene *p*‐coumarate (**18**): White amorphous solid; yield: 6.12% (22 mg, 0.14 mmol); mp: 164°C–165°C; *R*_f_ = 0.55 (hexane/EtOAc [8:2]); IR (KBr, *ʋ*_max_, cm^−^^1^): 3293, 3056, 2954, 1737, 1629, 1603, 1439, 1284 and 1168; ^1^H NMR (500 MHz, DMSO‐d_6_): δH 7.94‐7.91 (*m*, 4H), 7.63 (*d, J* = 15.00 Hz, 1H), 7.56 (*d*, *J* = 9.0 Hz, 2H), 7.53–7.51 (*m*, 3H), 6.80 (*d, J* = 9.0 Hz, 2H), 6.48 (*d*, *J* = 15.00 Hz, 1H), 5.37 (*s*, 2H); ^1^ ^3^C NMR (100 MHz, DMSO‐d_6_): δC 166.5, 159.9, 145.2, 134.0, 132.7, 132.6, 130.4, 128.1, 127.8, 127.6, 126.7, 126.4, 126.2, 125.9, 125.1, 115.8, 113.9, 65.6; HRMS (MALDI) calculated for C_20_H_16_O_3_ [M + Na]^+^: 327.0996, found: 327.1008.

### 4.5 Preparation of Compound **19**

Hexyl *p*‐coumarate (0.1 g; 0.40 mmol) in acetone (4 mL) was heated under reflux in the presence of potassium carbonate (1.18 mmol) and 1‐bromopropane (0.48 mmol) until complete reaction (16 h). The solvent was evaporated under reduced pressure, and 10 mL of water was added to the solution. The solution was then extracted with dichloromethane (3 × 10 mL). The organic phase was washed with water (10 mL), dried over anhydrous sodium sulfate, and concentrated under reduced pressure. The compound was isolated by CC on a silica gel 60 using hexane and ethyl acetate (8:2) as eluents [40].

Hexyl (2*E*)‐3‐(4‐propoxyphenyl)prop‐2‐enoate (**19**): Transparent liquid; yield: 64% (143 mg, 0.49 mmol); *R*_f_ = 0.7 (hexane/EtOAc [8:2]); IR (KBr, *ν*_max_, cm^−^^1^): 2963, 1733, 1638, 1605, 1469, 1255 and 1169; ^1^H NMR (400 MHz, CDCl_3_): δH 7.63 (*d*, *J* = 16.00 Hz, 1H), 7.46 (*d*, *J* = 8.0 Hz, 2H), 6.89 (*d*, *J* = 8.0 Hz, 2H), 6.30 (*d, J* = 16.00 Hz, 1H), 4.18 (*t*, *J* = 6.00 Hz, 2H), 3.94 (*t*, *J* = 6.00 Hz, 2H), 1.81 (*sext*, *J* = 6.76 Hz, 2H), 1.69 (*quint*, *J* = 6.56 Hz, 2H), 1.38–1.33 (*m*, 6H), 1.04 (*t*, *J* = 6.70 Hz, 3H), 0.90 (*t*, *J* = 6.92 Hz, 3H); ^1^ ^3^C NMR (100 MHz, CDCl_3_): δC 168, 161.1, 144.5, 129.8, 127.2, 115.8, 115.0, 69.8, 64.8, 31.6, 29.0, 25.9, 22.7, 22.6, 14.2, 10.7; HRMS (MALDI) calculated for C_18_H_26_O_3_ [M + H]^+^: 291.1960, found 291.1954.

### 4.6 Preparation of Compound **20**

Hexyl *p*‐coumarate (0.2 g; 0.80 mmol) in acetic anhydride (0.8 mL; 8.54 mmol) and pyridine (0.2 mL; 2.49 mmol) was stirred at room temperature for 24 h and monitored by TLC. The solvent was evaporated under reduced pressure, and 10 mL of water was added. The solution was then extracted with ethyl acetate (3 × 10 mL) and washed with 5% sodium bicarbonate (10 mL) and water (10 mL). The organic phase was dried over anhydrous sodium sulfate and concentrated under reduced pressure. The product was isolated by CC on a silica gel 60 using hexane and ethyl acetate (8:2) as eluents [41].

Hexyl (2*E*)‐3‐[4‐(acetyloxy)phenyl]prop‐2‐enoate (**20**): Colorless oil; yield: 60% (137 mg, 0.47 mmol); *R*_f_ = 0.64 (hexane/EtOAc [8:2]); IR (KBr, *ʋ*_max_, cm^−^^1^): 2932, 1769, 1712, 1638, 1603, 1459, 1282, and 1166; ^1^H NMR (400 MHz, CDCl_3_): δH 7.65 (*d*, *J* = 16.00 Hz, 1H), 7.54 (*d*, *J* = 8.00 Hz, 2H), 7.12 (*d*, *J* = 8.00 Hz, 2H), 6.39 (*d*, *J* = 16.00 Hz, 1H), 4.19 (*t*, *J* = 6.76 Hz, 2H), 2.31 (*s*, 3H), 1.68 (*m*, 2H), 1.43–1.30 (*m*, 6H), 0.90 (*t*, *J* = 6.92 Hz, 3H); ^1^ ^3^C NMR (100 MHz, CDCl_3_): δC 169.2, 167.2, 152.0, 143.6, 132.7, 129.6, 122.4, 118.6, 65.3, 31.7, 29.1, 25.7, 22.6, 21.3, 14.2; HRMS (MALDI) calculated for C_17_H_22_O_4_ [M + H]^+^: 291.1596; found 291.1590.

### 4.7 Preparation of Compounds **21–22**

Hexyl *p*‐coumarate (0.1 g; 0.40 mmol) was dissolved in 10% sodium hydroxide (0.6 mL). The acid chloride was added dropwise (0.40 mmol). The mixture was stirred at room temperature for 1 h and monitored by TLC. The solvent was evaporated under reduced pressure, and 10 mL of water was added. The solution was extracted with ethyl acetate (3 × 10 mL) and washed with 5% sodium bicarbonate (10 mL) and water (10 mL). The organic phase was dried over anhydrous sodium sulfate and concentrated under reduced pressure. The product was isolated by CC on a silica gel 60 using hexane and ethyl acetate (8:2) as eluents [41, 42].

4‐[(1*E*)‐3‐(Hexyloxy)‐3‐oxoprop‐1‐en‐1‐yl]phenyl benzoate (**21**): White solid; mp: 65–66°C; yield: 39% (111 mg, 0.31 mmol); *R*_f_ = 0.7 (hexane/EtOAc [8:2]); IR (KBr, *ν*_max_, cm^−^^1^): 2958, 1729, 1634, 1599, 1454, 1269, and 1161; ^1^H NMR (400 MHz, CDCl_3_): δH 8.19‐8.17 (*m* 2H), 7.67 (*d*, *J* = 16.00 Hz, 1H), 7.64‐7.62 (*m*, 1H), 7.61‐7.57 (*m*, 2H), 7.56‐7.50 (*m*, 2H), 7.25‐7.22 (*m*, 2H), 6.41 (*d*, *J* = 16.00 Hz, 1H), 4.19 (*t*, *J* = 6.80 Hz, 2H), 1.67 (*quint, J* = 6.80 Hz, 2H), 1.33–1.31 (*m*, 6H), 0.89 (*t*, *J* = 6.92 Hz, 3H); ^1^ ^3^C NMR (100 MHz, CDCl_3_): δC: 167.1, 165.1, 152.5, 143.6, 133.9, 132.4, 130.4, 129.4, 129.3, 128.8, 122.4, 118.7, 65.0, 31.6, 28.9, 25.8, 22.7, 14.3; HRMS (MALDI) calculated for C_22_H_24_O_4_ [M + Na]^+^: 375.1572; found: 375.1255.

4‐[(1*E*)‐3‐(Hexyloxy)‐3‐oxoprop‐1‐en‐1‐yl]phenyl 3‐bromobenzoate (**22**): White solid; mp: 40°C–41°C; yield: 52% (179.5 mg, 0.41 mmol); TLC (8:2 hexane/EtOAc), *R*_f_ = 0.74; IR (KBr, *ʋ*_max_, cm^−^^1^): 3067, 2930, 1741, 1711, 1636, 1599, 1472, 1290, 1176, 1062; ^1^H NMR (400 MHz, CDCl_3_): δH 8.33 (*t*, *J* = 1.6 Hz, 1H), 8.12 (*ddd*, J = 7.8, 1.56, 1.12 Hz, 1H), 7.77 (*ddd*, *J* = 8.00, 2.00, 1.10 Hz, 1H), 7.68 (*d*, *J* = 16.00 Hz, 1H), 7.59 (*d*, *J* = 8.50 Hz, 2H), 7.40 (*t*, *J* = 7.80 Hz, 1H), 7.24 (*d*, *J* = 8.50 Hz, 2H), 6.42 (*d*, *J* = 16.00 Hz, 1H), 4.21 (*t*, *J* = 6.76 Hz, 2H), 1.71 (*quint*, *J* = 6.80 Hz, 2H), 1.45–1.30 (*m*, 6H), 0.88 (*t*, *J* = 6.92 Hz, 3H); ^1^ ^3^C NMR (100 MHz, CDCl_3_): δC 167.1, 163.7, 152.2, 143.5, 136.9, 133.3, 132.7, 131.3, 130.3, 129.4, 128.9, 122.9, 122.3, 118.9, 65.0, 31.6, 28.8, 25.8, 22.7, 14.2; HRMS (MALDI) calculated for C_22_H_23_BrO_4_ [M + H]^+^: 431.0857, found: 431.0851.

### 4.8 Microtiter Enzyme Assays for AChE and BChE Inhibition

The derivatives were tested for their AChE and BChE inhibitory activities using a slightly modified spectrophotometric method based on Ellman et al. [43]. Briefly, add 140 µL of sodium phosphate buffer (pH 8.0), 10 µL of DTNB, 20 µL of test solution, and 20 µL of electric eel AChE (Sigma, EC number 3.1.1.7, 2.5 mU) or horse serum BChE (Sigma, EC number 3.1.1.8, 0.1 U), were added to a 96‐well microplate using an automatic multichannel pipette (Gilson Pipetman, France) and incubated for 10 min at 25°C. The reaction was initiated by adding 10 µL of acetylthiocholine iodide/butyrylthiocholine chloride. Hydrolysis was monitored by the formation of the yellow 5‐thio‐2‐nitrobenzoate anion at 412 nm using a 96‐well microplate reader (VersaMax Molecular Devices, USA). Measurements and calculations were evaluated using the Softmax PRO 4.3.2.LS software program. The percentage of AChE/BChE inhibition was determined by comparing the sample rates to blank rates (ethanol in phosphate buffer, pH 8.0) using the formula *I*% = 100 − [(*A*_1_/*A*_2_) × 100], where *A*_1_ is the absorbance of the sample and *A*_2_ is the average absorbance of the control at 412 nm. Results are expressed as mean ± S.D. of triplicate experiments. Galantamine was used as a reference anticholinesterase drug.

### 4.9 Ligand‐Binding Energy Calculation

3‐Dimensional crystal structures of AChE and BChE (4EY7 and 5DYW) were retrieved from the Protein Data Bank (PDB). Protein preparation wizard module of the Maestro of Schrödinger software was used to prepare the structures for the simulations. In the process of the preparation, the missing hydrogen atoms of the amino acids and any missing atoms of the side chains were added to the structures, bond orders inside the proteins were correctly assigned, the disulfide bonds were created, the water molecules beyond 5 Å from HET groups were deleted. The biological pH was set up using PROPKA, and restrained minimization for removing the clashes between the atoms was carried out using the OPLS3 force field. Structures of the ligands were downloaded from the PubChem server, and their structural energies were minimized to achieve a stable configuration. The positions of the simulation grid boxes were determined based on coordinates of the co‐crystal ligands binding to the proteins. The ligand binding energies inside the two proteins were estimated using the induced fit docking (IFD) method, which is able to keep atoms of the ligands and active site amino acids flexible during simulations and would generate more potential poses for the ligands inside the binding domains—and the results are presented as separate free binding energy for each pose [44, 45, 46].

## Author Contributions

**Susiany Pereira Lopes, Jeremias Justo Emídio, and Allana Brunna Sucupira Duarte**: Investigation and writing – original draft preparation. **Ramin Ekhteiari Salmas, Fatma Sezer Senol Deniz, and Ilkay Erdogan Orhan**: methodology. **Ilkay Erdogan Orhan and Fatma Sezer Senol Deniz**: data curation. **Damião Pergentino de Sousa**: writing – review and editing, and supervision.

## Funding

This research was funded by the Brazilian agencies Conselho Nacional de Desenvolvimento Científico e Tecnológico (CNPq; grant number: 306661/2016‐0, and Coordenação de Aperfeiçoamento de Pessoal de Nível Superior (CAPES).

## Conflicts of Interest

The authors declare no conflicts of interest.

## Supporting information




**Supporting File 1**: cbdv71128‐sup‐0001‐SuppMat.docx

## Data Availability

The authors declare that most of the data in this article are available in the dissertation free of charge from https://repositorio.ufpb.br/jspui/handle/123456789/26318.
