# Supplementary material for: Synthesis of p‐Coumarates With Potential Anti‐Alzheimer's Action: Enzyme Inhibition and In Silico Studies
Source: Chem Biodivers. 2026 Apr 1;23(4):e03857. doi: 10.1002/cbdv.202503857 (PMC13040117; doi:10.1002/cbdv.202503857)
Supplement: Supplementary file 1 — Supporting File 1: cbdv71128‐sup‐0001‐SuppMat.docx [file CBDV-23-e03857-s001.docx]

Synthesis of *p*-Coumarates with Potential Anti-Alzheimer's Action: Enzyme Inhibition and in silico Studies

Susiany Pereira Lopes ^1^, Jeremias Justo Emídio^1^, Allana Brunna Sucupira Duarte^1^, Ilkay Erdogan Orhan^2,3^, Fatma Sezer Senol Deniz^3,4^, Ramin Ekhteiari Salmas^5^, Damião Pergentino de Sousa^1,^*

1. Department of Pharmaceutical Sciences, Federal University of Paraíba, João Pessoa, CEP 58051-970, Paraíba, Brazil [susiany_lopes@hotmail.com](mailto:susiany_lopes@hotmail.com); [jeremiasjusto@gmail.com](mailto:jeremiasjusto@gmail.com); allanabrunna@gmail.com
2. Department of Pharmacognosy, Faculty of Pharmacy, Lokman Hekim University, Ankara 06510, Türkiye; [ilkay.erdoganorhan@lokmanhekim.edu.tr](mailto:ilkay.erdoganorhan@lokmanhekim.edu.tr)
3. Inovabella Biotechnology and R&D Industry and Trade Corp., LHUSTEK, 06510 Ankara, Türkiye
4. Department of Pharmacognosy, Faculty of Pharmacy, Gazi University, Ankara 06330, Türkiye; [fssenol@gazi.edu.tr](mailto:fssenol@gazi.edu.tr)
5. Department of Chemistry, Britannia House, King's College London, UK; ramin.ekhteiari_salmas@kcl.ac.uk

*****Correspondence: [damiao_desousa@yahoo.com.br](mailto:damiao_desousa@yahoo.com.br)

**Supporting Information**

**Chemical characterization of compounds 1-22**

**(*E*)-**
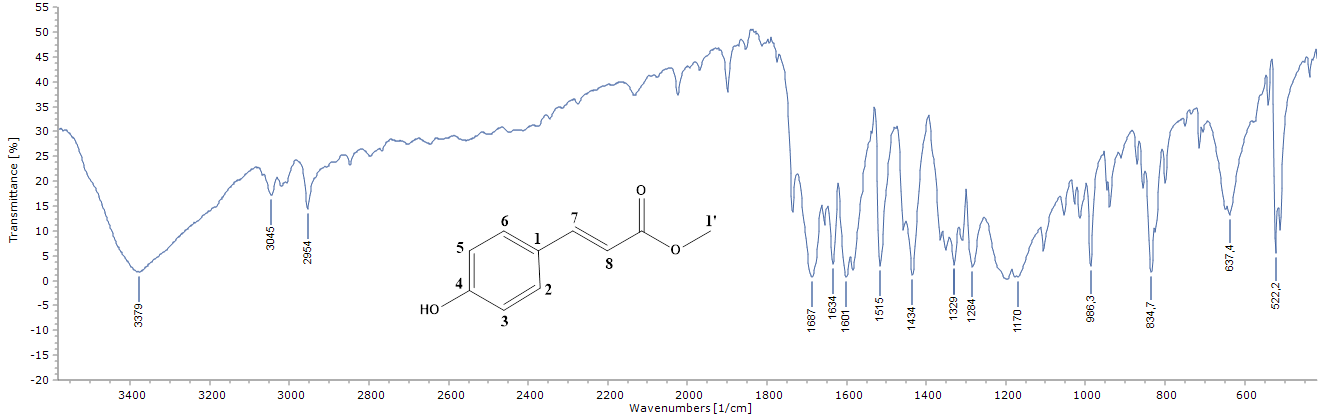
**Methyl *p*-coumarate** (**1**):White amorphous solid; yield: 87.98% (95.5 mg; 0.53 mmol); melting point: 135–136 °C (lit. 129–132 °C, Allegretta et al., 2015); Rf = 0.52 (hexane/EtOAc [7:3]); **IR ʋmax** **(KBr, cm⁻¹):** 3379, 3045, 2954, 1687, 1634, 1601, 1284 and 1170; **¹H NMR (400 MHz, DMSO-d₆):** *δ*H 7.56 (*d*, *J* = 16 Hz, 1H), 7.54 (*d*, *J* = 8.0 Hz, 2H), 6.79 (*d*, *J* = 8.0 Hz, 2H), 6.38 (*d*, *J* = 16 Hz, 1H), 3.69 (*s*, 3H); **¹³C NMR (100 MHz, DMSO-d₆):** δC 167.1, 159.9, 144.9, 130.4, 125.2, 115.8, 114.0, 51.3 (Allegretta et al., 2015; Khatkar et al., 2017; Lopes et al., 2019).

**Figure S1:** Infrared spectrum ʋmax (KBr, cm^-1^) of compound **1**

**Figure** **S2:** ^1^H NMR spectrum (400 MHz, CDCl_3_) of compound **1**

**Figure S3:** ^13^C NMR spectrum (APT, 100 MHz, CDCl_3_) of compound **1**

**(E)-Ethyl p-coumarate** (**2**): White solid; yield: 84.21% (295.8 mg; 1.53 mmol); melting point: 76–77 ºC (lit. 72–73 ºC, HIXSON et al., 2012); Rf = 0.55 (hexane/EtOAc [7:3]); **IR ʋmax (KBr, cm⁻¹):** 3286, 3026, 2985, 1683, 1634, 1604, 1440, 1280 and 1169; **¹H NMR (400 MHz, CDCl₃):** δH 7.63 (d, J = 16.00 Hz, 1H), 7.39 (d, J = 8.00 Hz, 2H), 6.87 (d, J = 8.00 Hz, 2H), 6.28 (d, J = 16.00 Hz, 1H), 4.26 (q, J = 8.00 Hz, 2H), 1.33 (t, J = 8.00 Hz, 3H); **¹³C NMR (100 MHz, CDCl₃):** δC 168.5, 158.7, 145.5, 130.2, 126.8, 116.1, 115.0, 61.0, 14.4 (HIXSON et al., 2012; HOSSEINI et al., 2016; KHATKAR et al., 2017; LOPES et al., 2019).


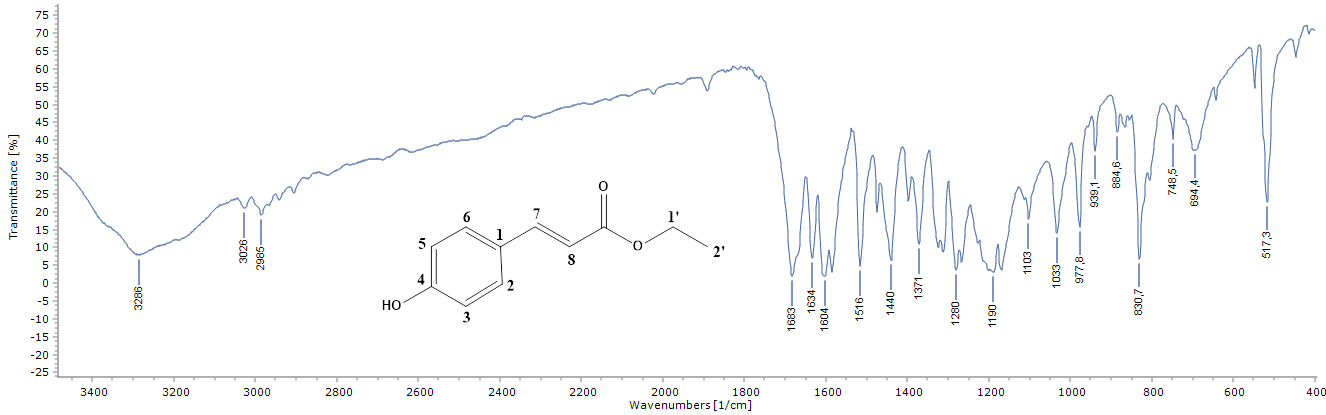


**Figure S4:** Infrared spectrum ʋmax (KBr, cm^-1^) of compound **2**

**Figure S5:** ^1^H NMR spectrum (400 MHz, CDCl_3_) of compound **2**

**Figure S6:** ^13^C NMR spectrum (APT, 100 MHz, CDCl_3_) of compound **2**

**(*E*)-Propyl *p*-coumarate** (**3**): White solid; yield 58.43% (220.2 mg; 1.06 mmol); melting point: 74–75 °C (lit. 148–150 °C, KHATKAR et al., 2017); Rf = 0.57 (hexane/EtOAc 7:3); **IR (KBr, νmax, cm⁻¹):** 3376, 3028, 2879, 1674, 1638, 1603, 1441, 1271 and 1171; **¹H NMR (400 MHz, CDCl₃):** *δ*H 7.63 (*d*, *J* = 16.00 Hz,, 1H), 7.42 (*d*, *J* = 8.00 Hz, 2H), 6.85 (*d*, *J* = 8.00 Hz, 2H), 6.30 (*d*, *J* = 16.00 Hz, 1H), 4.16 (*t*, *J* = 8.0 Hz, 2H), 1.73 (*m*, 2H), 0.99 (*t*, *J* = 8.0 Hz, 3H); **¹³C NMR (400 MHz, CDCl₃):** *δ*C 168.5; 158.3; 145.0; 130.2; 127.1; 116.0; 115.4; 66.5; 22.2; 10.8 (KHATKAR et al., 2017; LOPES et al., 2019).


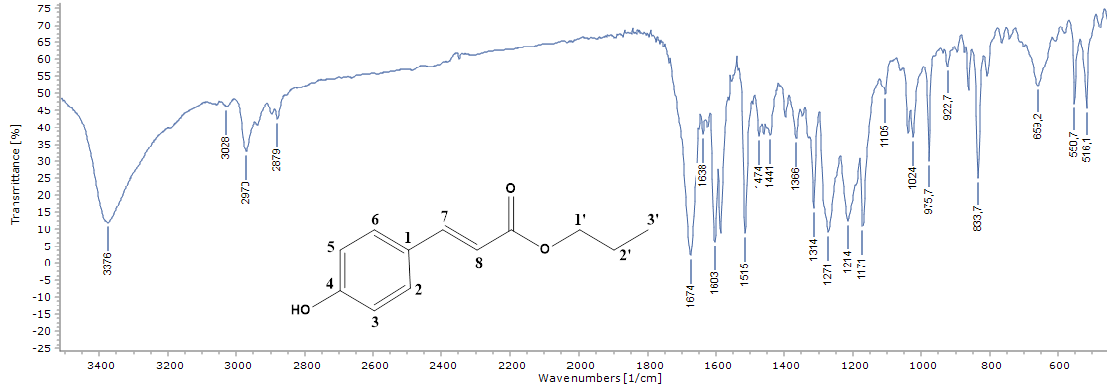


**Figure S7:** Infrared spectrum ʋmax (KBr, cm^-1^) of compound **3**

**Figure S8:** ^1^H NMR spectrum (400 MHz, CDCl_3_) of compound **3**

**Figure S9:** ^13^C NMR spectrum (APT, 100 MHz, CDCl_3_) of compound **3**

**(*E*)-Isopropyl *p*-coumarate** (**4):** White solid; yield: 60.73% (228.9 mg; 1.10 mmol); melting point: 71–72 ºC (lit. 71–73 ºC, KHATKAR et al., 2017); Rf = 0.57 (hexane/EtOAc [7:3]); **IR (KBr, ν_max, cm⁻¹):** 3272, 3046, 2939, 1675, 1629, 1605 1466, 1279 and 1172; **¹H NMR (400 MHz, CDCl₃):** δC 7.61 (*d*, *J* = 16.00 Hz, 1H), 7.38 (*d*, *J* = 8.00 Hz, 2H), 6.87 (*d*, *J* = 8.00 Hz, 2H), 6.27 (*d, J* = 16.00 Hz, 1H), 5.14 (*sept,* *J* = 8 Hz,1H), 1.31 (*d*, *J* = 8.00 Hz, 6H); **¹³C NMR (100 MHz, CDCl₃):** δC 168.1, 158.7, 145.0, 130.3, 126.8, 116.1, 115.6, 68.4, 22.2 (C-2′) (KHATKAR et al., 2017; LOPES et al., 2019).

**
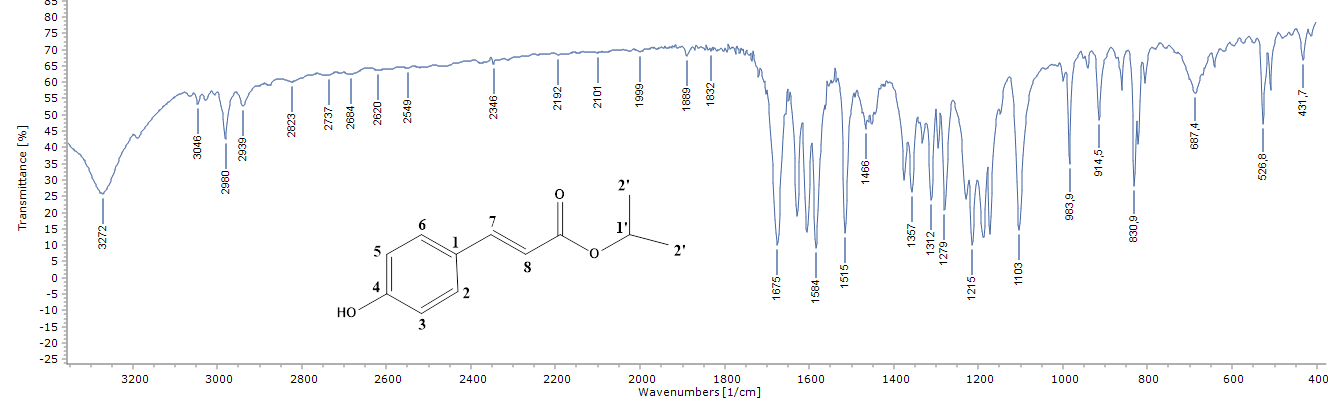
**

**Figure S10:** Infrared spectrum ʋmax (ATR, cm^-1^) of compound **4**

**Figure S11:** ^1^H NMR spectrum (400 MHz, CDCl_3_) of compound **4**

**Figure S 12:** ^13^C NMR spectrum (APT, 100 MHz, CDCl_3_) of compound **4**

**(*E*)-2-Methoxyethyl *p*-coumarate** (**5**)**:** White solid; yield: 42.76% (173.7 mg; 0.78 mmol); melting point: 96–97 ºC; Rf = 0.33 (hexane/EtOAc [7:3]); **IR (KBr, ʋmax, cm⁻¹):** 3260, 3043, 2959, 1696, 1655, 1609, 1447, 1266 and 1176; **¹H NMR (400 MHz, CDCl₃):** δC 7.60 (*d*, *J* = 16.00 Hz, 1H), 7.33 (*d, J* = 8.00 Hz, 2H), 6.81 (*d*, *J* = 8.00 Hz, 2H), 6.21 (*d*, *J* = 16.00 Hz, 1H), 4.37 (*t*, *J* = 4.00 Hz, 2H), 3.71 (*t*, *J* = 4.00 Hz, 2H), 3.46 (*s*, 3H); **¹³C NMR (100 MHz, CDCl₃):** δC 167.8, 158.8, 145.6, 130.1, 126.5, 116.1, 114.6, 70.8, 63.2, 58.8 (KHATKAR et al., 2017; LOPES et al., 2019).


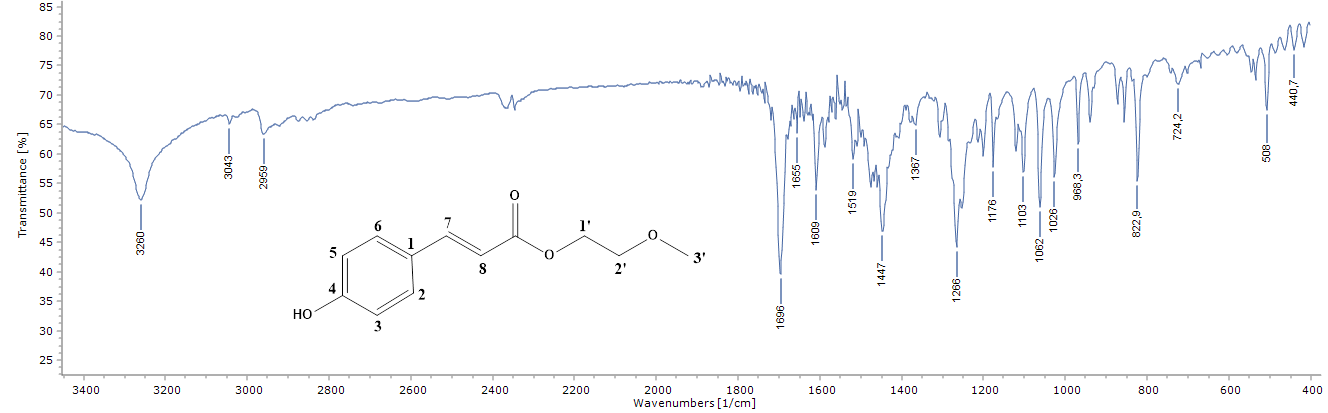


**Figure S 13:** Infrared spectrum ʋmax (KBr, cm^-1^) of compound **5**

**Figure S**14: ^1^H NMR spectrum (400 MHz, CDCl_3_) of compound **5**

**Figure S15:** ^13^C NMR spectrum (APT, 100 MHz, CDCl_3_) of compound **5**

**(*E*)-Butyl *p*-coumarate** (**6**)**:** Amorphous brown solid; yield 38% (154.2 mg; 0.70 mmol); Rf = 0.65 (hexane/EtOAc [7:3]); **IR ʋmax (KBr, cm⁻¹):** 3377, 3024, 2873, 1683, 1634, 1604, 1474, 1280, and 1172. **¹H NMR (400 MHz, CDCl₃):** δH 7.62 (*d*, *J* = 16.0 Hz, 1H); 7.42 (*d*, *J* = 8.00 Hz, 2H); 6.85 (*d*, *J* = 8.00 Hz, 2H); 6.30 (*d*, *J* = 16.00 Hz, 1H); 4.20 (*t*, *J* = 8.00 Hz, 3H); 1.70-1.45 (*m*, 2H); 1.44-1.24 (*m*, 2H); 0.96 (*t*, *J* = 8.00 Hz, 3H); **¹³C NMR (100 MHz, CDCl₃):** δC 168.5, 158.5, 145.2, 130.2, 126.8, 116.1, 115.2, 64.8, 30.8, 19.3, 14.0 (KHATKAR et al., 2017; LOPES et al., 2019).

**
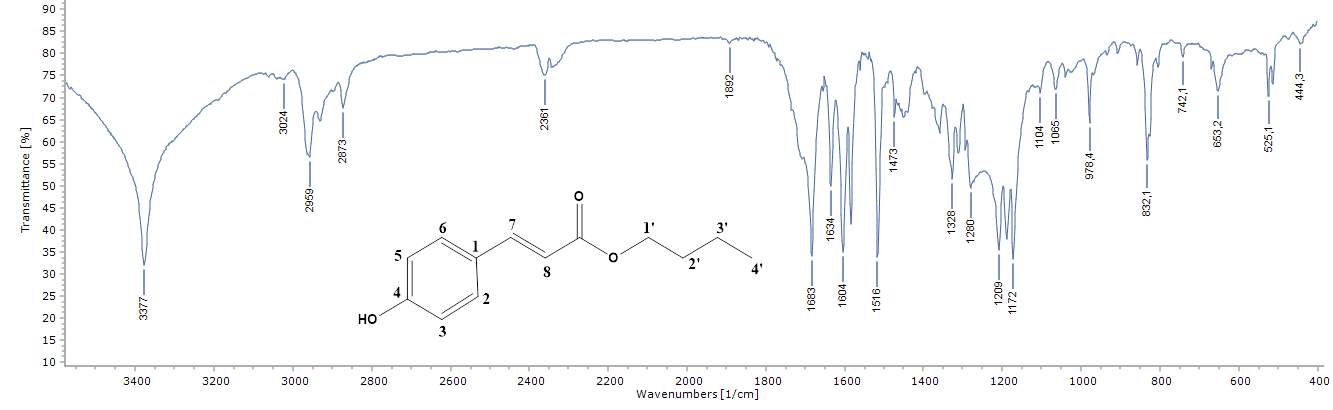
**

**Figure S16:** Infrared spectrum ʋmax (ATR, cm^-1^) of compound **6**

**Figure S 17:** ^1^H NMR spectrum (400 MHz, CDCl_3_) of compound **6**

**Figure S18:** ^13^C NMR spectrum (APT, 100 MHz, CDCl_3_) of compound **6**

**(E)-Pentyl p-coumarate** (**7**)**:** Amorphous amber-colored solid with a yield of 44.09% (188.8 mg; 0.80 mmol); Rf = 0.60 (hexane/EtOAc [7:3]); **IR (KBr, ν_max, cm⁻¹):** 3400, 3027, 2872, 1685, 1632, 1605, 1445, 1268 and 1170. **¹H NMR (400 MHz, CDCl₃):** δC 7.65 (d, J = 16.0 Hz, 1H), 7.42 (d, J = 8.0 Hz, 2H), 6.88 (d, J = 8.00 Hz, 2H), 6.32 (d, J = 16.00Hz, 1H), 4.22 (t, J = 8.00 Hz, 2H), 1.73-1.67 (m, 2H), 1.42–1.38 (m, 4H), 0.94 (t, J = 7.20 Hz, 3H); **¹³C NMR (100 MHz, CDCl₃):** δC 168.4, 158.5, 145.1, 130.2, 126.9, 116.1, 115.2, 65.1, 28.5, 28.2, 22.5, 14.1 (KHATKAR et al., 2017; LOPES et al., 2019).

**
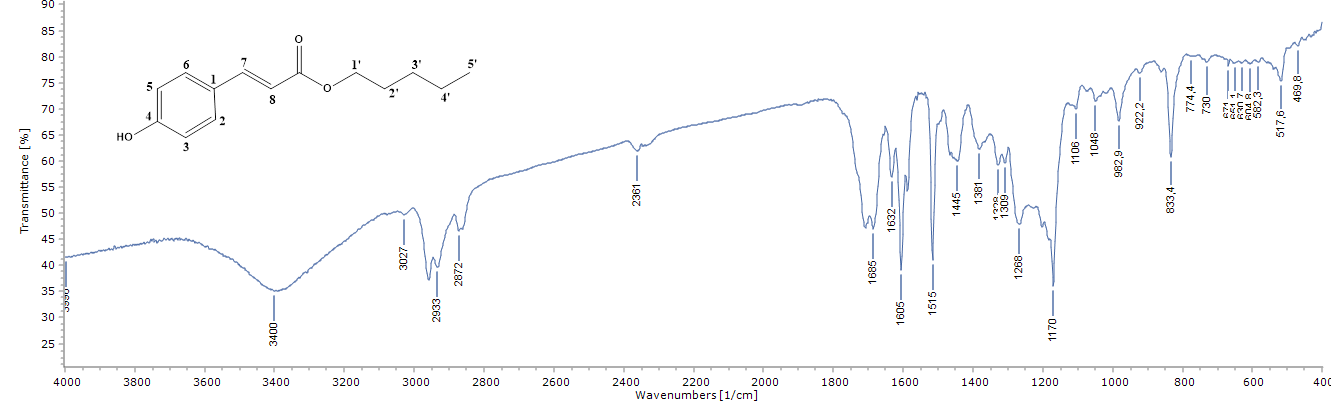
**

**Figure S19:** Infrared spectrum ʋmax (KBr, cm^-1^) of compound **7**

**Figure** **S20:** ^1^H NMR spectrum (400 MHz, CDCl_3_) of compound **7**

**Figure S 21:** ^13^C NMR spectrum (APT, 100 MHz, CDCl_3_) of compound **7**

**(*E*)-Isopentyl *p*-coumarate** (**8**)**:** Brown solid; yield: 79.36% (339.8 mg; 1.45 mmol); melting point: 111–112 °C (lit. 151–153 °C, KHATKAR et al., 2017); Rf = 0.66 (hexane/EtOAc [7:3]); **IR (KBr, ʋmax, cm⁻¹):** 3380, 3027, 2873, 1687, 1637, 1604, 1437, 1277 and 1166; **¹H NMR (400 MHz, CDCl₃):** δH 7.64 (*d*, *J* = 16.00 Hz, 1H), 7.39 (*d*, *J* = 8.00 Hz, 2H), 6.86 (*d, J* = 8.00 Hz, 2H), 6.31 (*d*, *J* = 16.00 Hz, 1H), 4.23 (*t*, *J* = 8.00 Hz, 2H), 1.72 (*sept*, *J* = 6.00 Hz, 1H), 1.65-1.58 (*m,* 2H) 0.95 (*d*, *J* = 6.00 Hz, 6H); **¹³C NMR (100 MHz, CDCl₃):** δC 168.6 , 158.6, 145.1, 130.2, 126.8, 116.1, 115.2, 63.6, 37.5, 25.3, 22.6 (KHATKAR et al., 2017; LOPES et al., 2019).


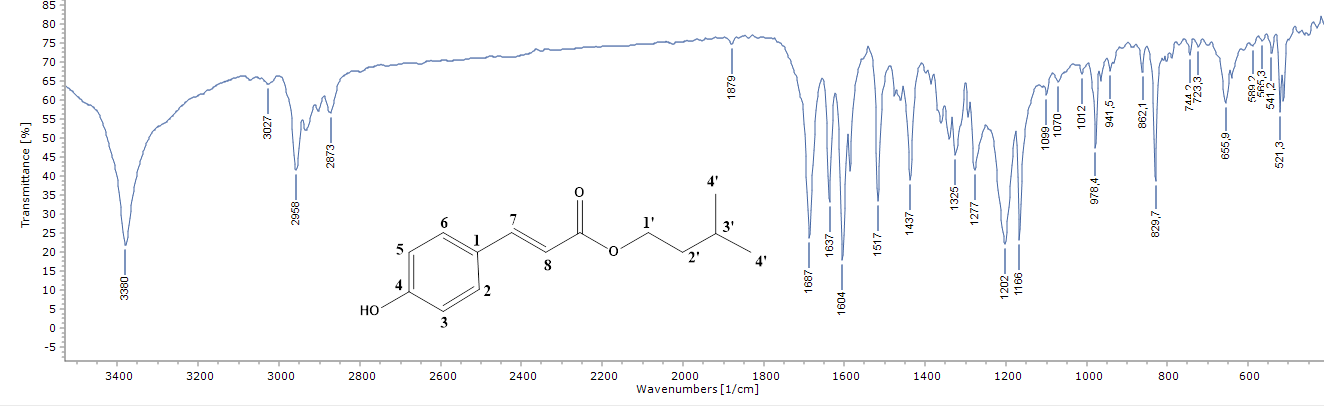


**Figure S 22:** Infrared spectrum ʋmax (KBR, cm^-1^) of compound **8**

**Figure S23:** ^1^H NMR spectrum (400 MHz, CDCl_3_) of compound **8**

**Figure** **S 24:** ^13^C NMR spectrum (APT, 100 MHz, CDCl_3_) of compound **8**

**(E)-Hexyl p-coumarate** (**9**)**:** White solid; yield: 36.88% (167.4 mg; 0.67 mmol); melting point: 42–43 ºC (lit. 160 ºC, PATEL et al., 2011); Rf = 0.47 (hexane/EtOAc [8:2]); **IR (KBr, ʋmax, cm⁻¹):** 3383, 3026, 2857, 1675, 1625, 1604, 1472, 1276 and 1170; **¹H NMR (400 MHz, CDCl₃):** δH: 7.63 (d, J = 16.00 Hz, 1H), 7.41 (d, J = 8.00 Hz, 2H), 6.87 (d, J = 8.00 Hz, 2H), 6.28 (d, J = 16.00 Hz, 1H), 4.19 (t, J = 8.00 Hz, 2H), 1.71-1.70 (m, 2H), 1.41–1.32 (m, 6H), 0.89 (t, J = 6.00 Hz,, 3H**); ¹³C NMR (100 MHz, CDCl₃):** δC 168.3, 158.4, 145.0, 130.1, 127.0, 116.1, 115.4, 65.1, 31.6, 28.8, 25.8, 22.7, 14.1 (KHATKAR et al., 2017; LOPES et al., 2019).

**
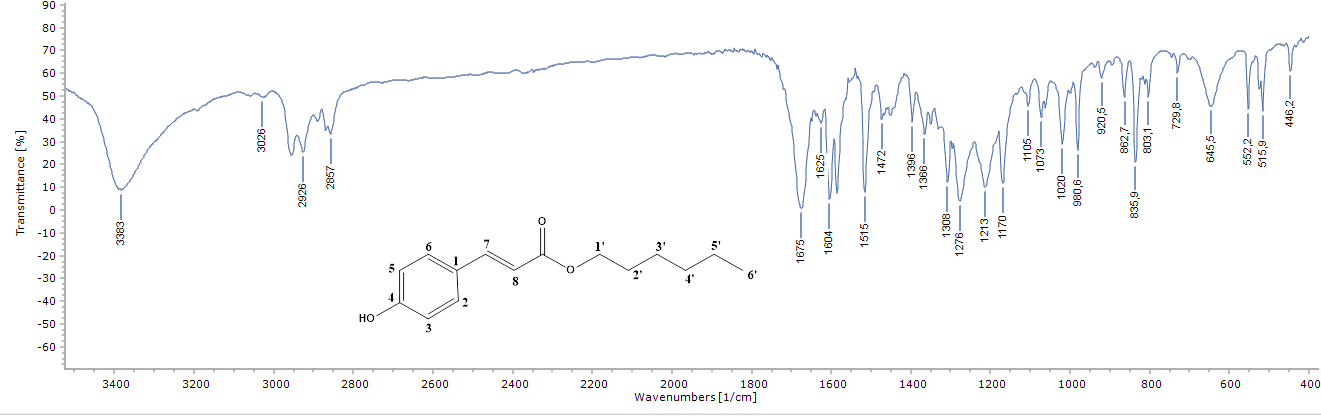
**

**Figure S 25:** Infrared spectrum ʋmax (ATR, cm^-1^) of compound **9**

**Figure S26:** ^1^H NMR spectrum (400 MHz, CDCl_3_) of compound **9**

**Figure S27:** ^13^C NMR spectrum (APT, 100 MHz, CDCl_3_) of compound **9**

**(*E*)-Decyl *p*-coumarate** (**10**)**:** White amorphous solid; yield: 16.44% (122 mg; 0.40 mmol); melting point: 68–69 ºC (lit. 62–64 ºC, NISHIMURA et al., 2009); Rf = 0.75 (hexane/EtOAc [8:2]); **IR (KBr, ʋmax, cm⁻¹):** 3383, 3032, 2926, 2848, 1678, 1625, 1604, 1474, 1273 and 1172; **¹H NMR (400 MHz, CDCl₃)**: δH 7.63 (*d*, *J* = 16.00 Hz, 1H), 7.41 (*d*, *J* = 8.00 Hz, 2H), 6.86 (*d,* *J* = 8.00 Hz, 2H), 6.29 (*d*, *J* = 16.00 Hz, 1H), 4.20 (*t, J* = 6.00 Hz, 2H), 1.70-1.40 (*m*, 2H), 1.39–1.26 (*m*, 14H), 0.87 (*t*, *J* = 6.00 Hz, 3H); **¹³C NMR (100 MHz, CDCl₃)**: δC 168.2, 158.3, 145.2, 130.1, 127.0, 116.1, 115.4, 64.9, 32.0, 29.7, 29.4, 28.8, 26.1, 22.8, 14.2 (NISHIMURA et al., 2009; HOSSEINI et al., 2016).

**
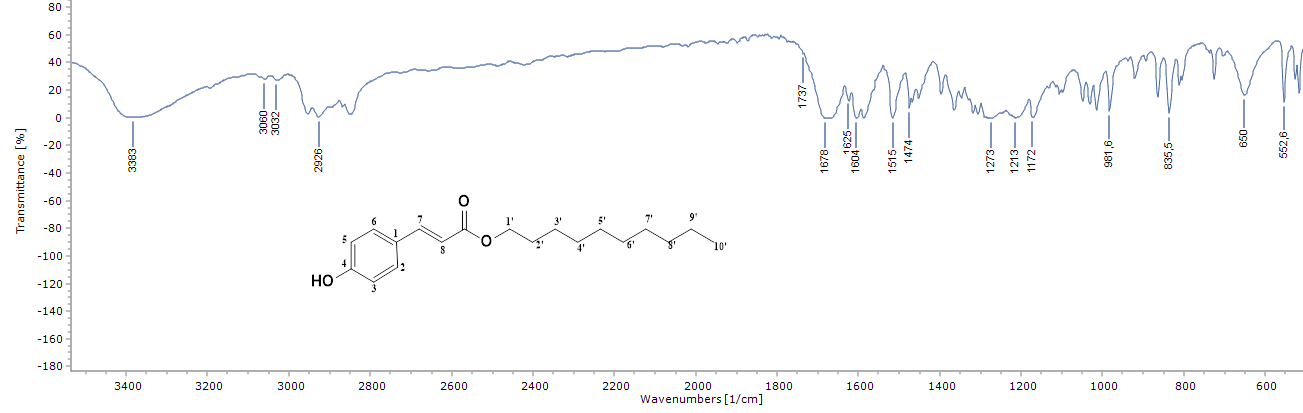
**

**Figure S28:** Infrared spectrum ʋmax (ATR, cm^-1^) of compound **10**

**Figure S29:** ^1^H NMR spectrum (400 MHz, CDCl_3_) of compound **10**.

**Figure S30**: ^13^C NMR spectrum (APT, 100 MHz, CDCl_3_) of compound **10**

**(*E*)-Dodecyl *p*-coumarate (11):** White solid; yield 29.55% (179.6 mg, 0.54 mmol); melting point 76–77 °C (lit. 73–75 °C, NISHIMURA et al., 2009); TLC (hexane/EtOAc 8:2), **Rf = 0.52; IR νmax (KBr, cm⁻¹):** 3381, 3027, 2848, 1674, 1623, 1603, 1473, 1275 and 1171; **¹H NMR (400 MHz, CDCl₃):** δ 7.63 (*d*, *J* = 16.00 Hz, 1H), 7.41 (*d*, *J* = 8.00 Hz, 2H), 6.87 (*d*, *J* = 8.00 Hz, 2H), 6.30 (*d*, *J* = 16.00 Hz, 1H), 4.20 (*t,* *J* = 6.72 Hz, 2H), 1.70 (*quint*, *J* = 6.72 Hz, 2H), 1.39–1.26 (*m*, 18H), 0.88 (*t*, *J* = 6.68 Hz, 3H); **¹³C NMR (100 MHz, CDCl₃):** 168.5, 158.5, 145.1, 130.0, 126.9, 116.0, 115.3, 64.9, 31.9, 29.7, 29.6, 29.6, 29.5, 29.4, 29.3, 28.7, 26.0, 22.7, 14.1 (HOSSEINI et al., 2016; LOPES et al., 2019).


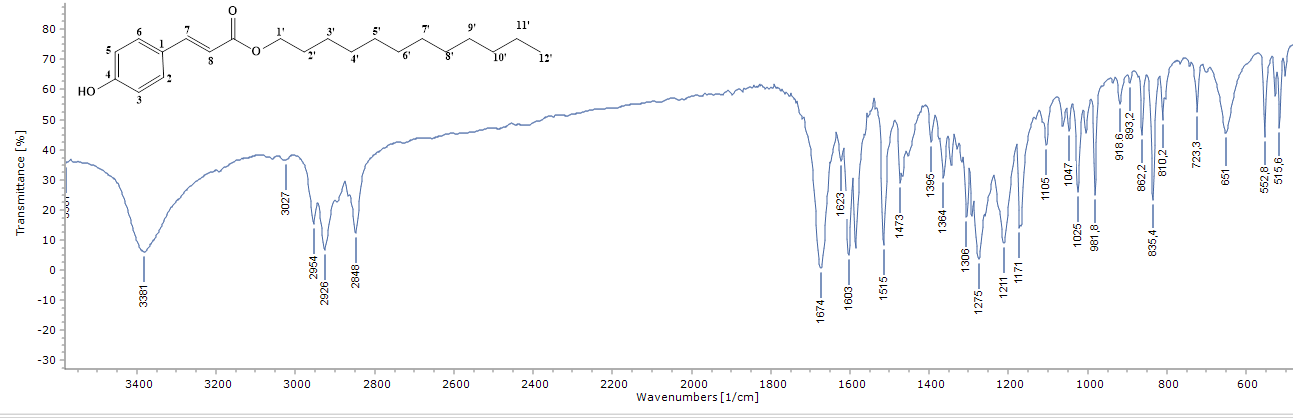


**Figure S31:** Infrared spectrum ʋmax (ATR, cm^-1^) of compound **11**

**Figure 32:** ^1^H NMR spectrum (400 MHz, CDCl_3_) of compound **11**

**Figure S33:** ^13^C NMR spectrum (APT, 100 MHz, CDCl_3_) of compound **11**

**(E)-4-Methylbenzyl p-coumarate** (**12**)**:** White amorphous solid; yield: 30% (147.2 mg; 0.54 mmol); melting point: 93–94 ºC (lit. 106–108 ºC, KIM et al., 2010); Rf = 0.34 (hexane/EtOAc [8:2]); **IR νmax (KBr, cm⁻¹):** 3291 , 3027, 2958, 1689, 1629, 1605, 1450, 1279 and 1164; **¹H NMR (400 MHz, CDCl₃)**: δH 7.66 (d, J = 16.00 Hz, 1H), 7.39 (d, J = 8.00 Hz, 2H), 7.31 (d, J = 8.00 Hz, 2H), 7.19 (d, J = 8.00 Hz, 2H), 6.85 (d, J = 8.00 Hz, 2H), 6.33 (d, J = 16.00 Hz, 1H), 5.22 (s, 2H), 2.37 (s, 3H); **¹³C NMR (400 MHz, CDCl₃):** δC 168.0, 158.3, 145.5, 138.3, 133.1, 130.2, 129.4, 128.6, 127.0, 116.1, 115.1, 66.6, 21.7 (KIM et al., 2010; LOPES et al., 2019).


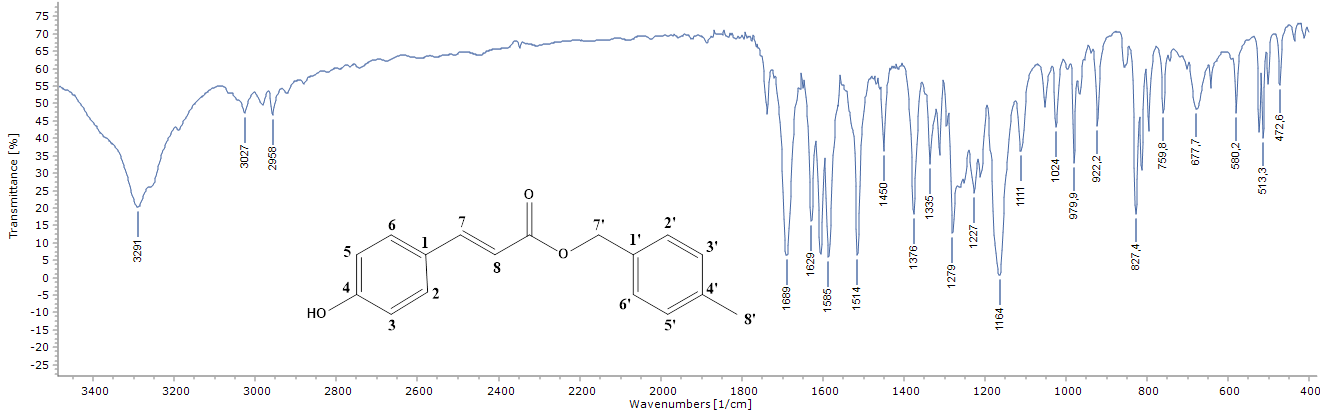


**Figure S34:** Infrared sprectrum ʋmax (ATR, cm^-1^) of compound **12**

**Figure S35:** ^1^H NMR spectrum (400 MHz, CDCl_3_) of compound **12**

**Figure S36:** ^13^C NMR spectrum (APT, 125 MHz, CDCl_3_) of compound **12**

*(E*)-4‑Isopropylbenzyl *p*‑coumarate (13): White amorphous solid; yield: 29.76% (161.2 mg, 0.54 mmol); melting point: 107–108 ^o^C; Rf = 0.39 (hexane/EtOAc [8:2]); IR ʋmax (KBr, cm⁻¹): 3232, 3031, 2960, 1709, 1638, 1605, 1455, 1226 and 1158; ¹H NMR (400 MHz, CDCl₃): δH 7.69 (*d*, *J* = 16.00 Hz, 1H), 7.42 (*d*, *J* = 8.00 Hz, 2H), 7.37 (*d*, *J* = 8.00 Hz, 2H), 7.27 (*d*, *J* = 8.00 Hz, 2H), 6.87 (*d*, *J* = 8.00 Hz, 2H), 6.35 (*d*, *J* = 16.00 Hz, 1H), 5.24 (*s*, 2H), 2.94 (*sept*, *J* = 6.92 Hz, 1H), 1.28 (*d*, *J* = 6.92 Hz, 6H); ¹³C NMR (100 MHz, CDCl₃): δC 167.9, 158.2, 149.3, 145.3, 133.5, 130.2, 128.6, 127.1, 126.8, 116.1, 115.2, 66.6, 34.2, 24.1 (Lopes et al., 2019).

**(*E*)- 4-isopropylbenzyl *p*-Coumarate** (**13**)**:**


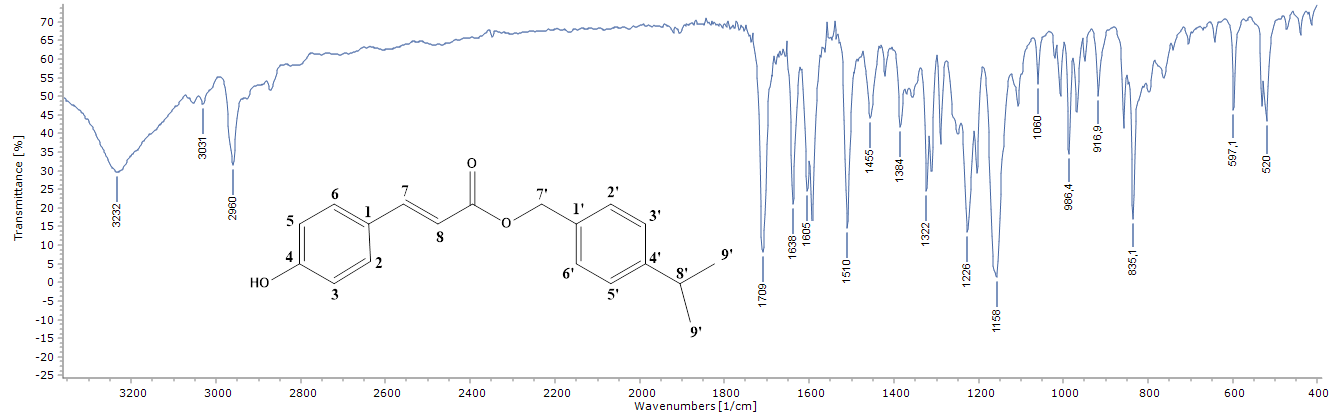


**Figure S37:** Infrared sprectrum ʋmax (KBr, cm^-1^) of compound **13**

**Figure38:** ^1^H NMR spectrum (400 MHz, CDCl_3_) of compound **13**

**Figure S39:** ^13^C NMR spectrum (APT, 100 MHz, CDCl_3_) of compound **13**

**(*E*)-4-Chlorobenzyl *p*-Coumarate** (**14**)**:** White amorphous solid; yield 30.84% (217 mg, 0.75 mmol); melting point: 111–112 °C (114–116 °C, KIM et al., 2010). Rf = 0.62 (hexane/EtOAc [8:2]); **IR νmax (KBr, cm⁻¹):** 3414, 3049, 2893, 1697, 1632, 1600, 1440, 1280, 1166 and 550.4; **¹H NMR (400 MHz, CDCl₃):** δH 7.67 (d, J = 16.00 Hz, 1H); 7.41 (*d, J* = 8.00 Hz, 2H); 7.34 (*m*, 4H); 6.85 (*d*, *J* = 8.00 Hz, 2H); 6.35 (*d, J* = 16.00 Hz, 1H); 5.21 (s, 2H); **¹³C NMR (100 MHz, CDCl₃):** 167.8, 158.4, 145.7, 134.6, 134.3, 130.3, 129.7, 128.9, 126.9, 116.1, 114.7, 65.7 (KIM et al., 2010).


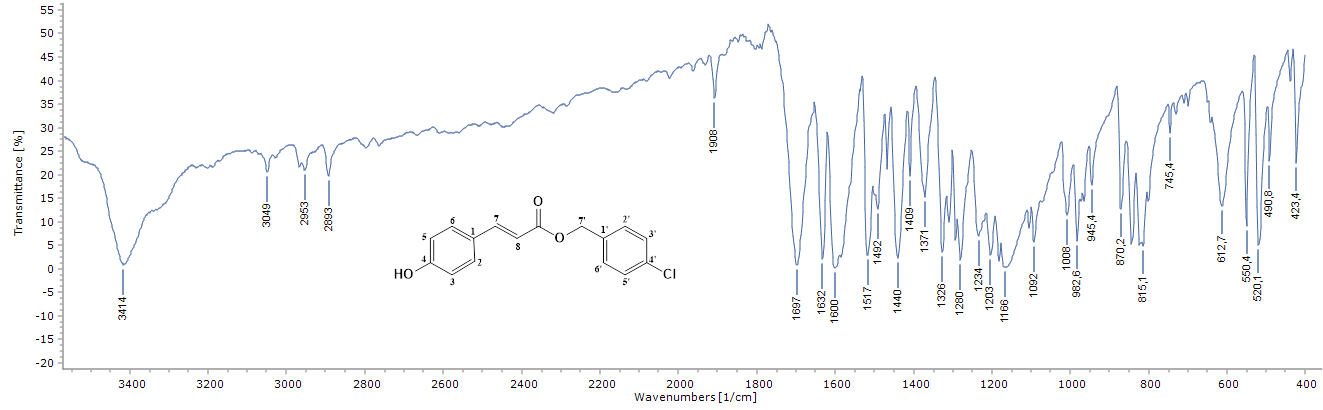


**Figure 41:** Infrared spectrum ʋmax (KBr, cm^-1^) of compound **14**

**Figure S42:** ^1^H NMR spectrum (400 MHz, CDCl_3_) of compound **14**

**Figure S43:** ^13^C NMR spectrum (APT, 100 MHz, CDCl_3_) of compound **14**

**(*E*)-4-Bromobenzyl *p*-Cumarate** (**15**)**:**


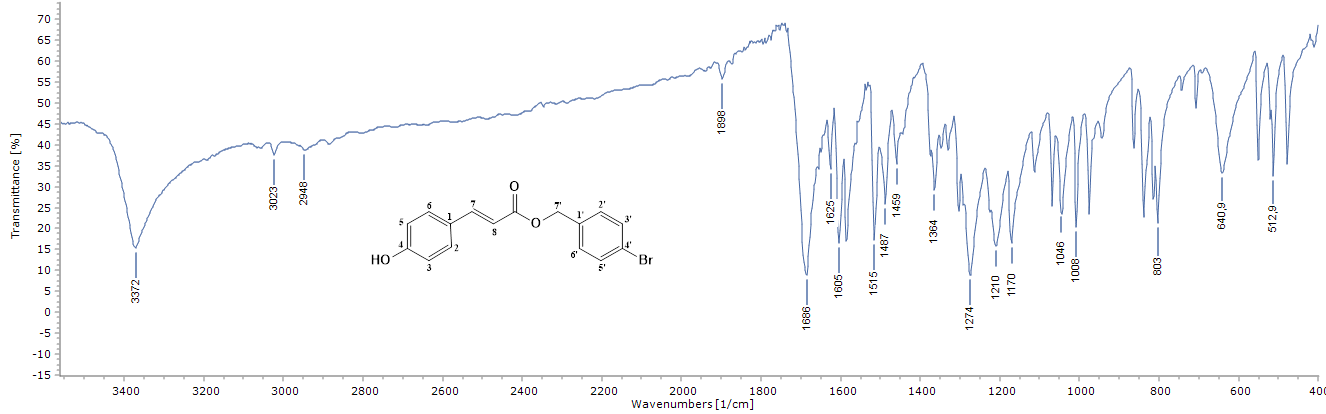


**Figure S44:** Infrared spectrum ʋmax (KBr, cm^-1^) of compound **15**

**Figure S45**: ^1^H NMR spectrum (400 MHz, CDCl_3_) of compound **15**

**Figure S46:** ^13^C NMR spectrum (APT, 100 MHz, CDCl_3_) of compound **15**


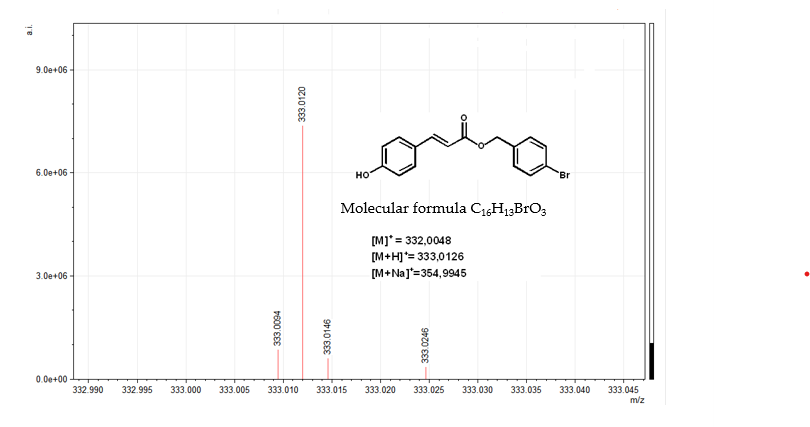


**Figure S47:** HRMS spectrum of compound **15**

**(*E*)-4-Methoxybenzyl *p*-Cumarate** (**16**)**:** White amorphous solid; yield: 10.83% (75 mg, 0.26 mmol); melting point: 82–83 ºC (lit. 90–92 ºC, KIM et al., 2010); Rf = 0.5 (hexane/EtOAc [8:2]); **IR νmax (KBr, cm⁻¹):** 3278, 3028, 2959, 1678, 1634, 1602, 1442, 1280 and 1171; **¹H NMR (400 MHz, CDCl₃):** δH 7.65 (*d*, *J* = 16.00 Hz, 1H), 7.39 (*d*, *J* = 8.00 Hz, 2H), 7.35 (*d*, *J* = 8.00 Hz, 2H), 6.91 (*d*, *J* = 8.00 Hz, 2H), 6.84 (*d*, *J* = 8.00 Hz, 2H), 6.31 (*d, J* = 16.00 Hz, 1H), 5.19 (*s*, 2H), 3.81 (*s*, 3H); **¹³C NMR (100 MHz, CDCl₃):** δC 167.9, 159.7, 158.4, 145.3, 130.3, 130.2, 128.3, 126.9, 116.1, 115.1, 114.1, 66.5, 55.5 (KIM et al., 2010).

**
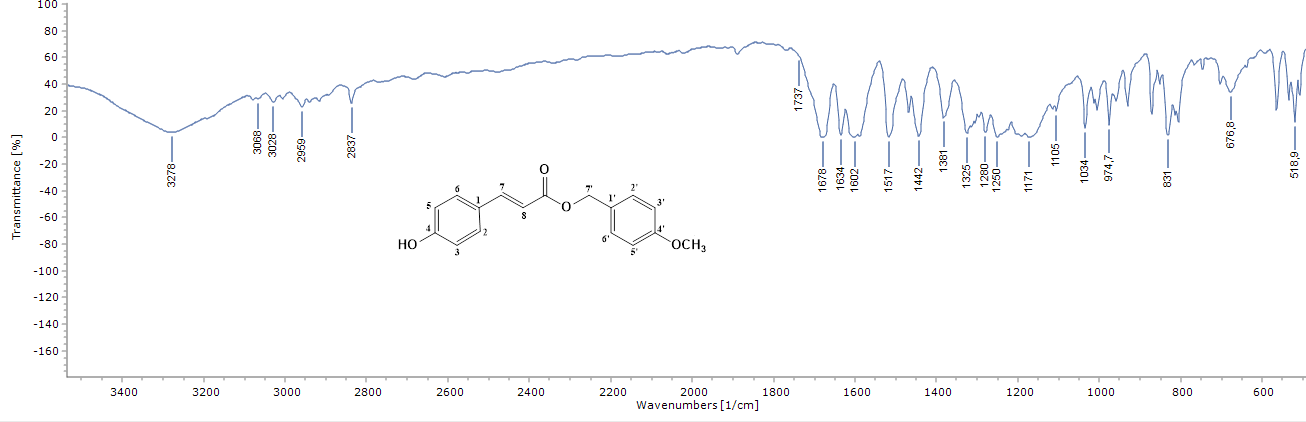
**

**Figure S48:** Infrared spectrum ʋmax (KBr, cm^-1^) of compound **16**

**Figure S49:** ^1^H NMR spectrum (400 MHz, CDCl_3_) of compound **16**

**Figure S50**: ^13^C NMR spectrum (APT, 100 MHz, CDCl_3_) of compound **16**

**Figure S51:** ^13^C NMR spectrum (APT, 100 MHz, CDCl_3_) of compound **16**

**(*E*)-Phenethyl *p*-coumarate** (**17**): White amorphous solid; yield: 6.64% (43.8 mg; 0.16 mmol); melting pointe: 90–91 ºC (lit. 90–92 ºC, KIM et al., 2010). Rf = 0.62 (hexane/EtOAc [8:2]); **IR (KBr, νmax, cm⁻¹):** 3389, 3025, 2923, 1685, 1636, 1595, 1457, 1280 and 1171; **¹H NMR (500 MHz, CDCl₃):** δH 7.31 (*d*, *J* = 15.00 Hz, 1H), 7.09 (*d*, *J* = 10.00 Hz, 2H), 7.00 (*m*, 2H), 6.95 (*m*, 3H), 6.54 (*d*, *J* = 10.00 Hz, 2H), 5.98 (*d*, *J* = 15.00 Hz, 1H), 4.12 (*t*, *J* = 10.00 Hz, 2H), 2.71 (*t*, *J* = 10.00 Hz, 2H); **¹³C NMR (100 MHz, CDCl₃):** δ 167.9, 158.2, 145.1, 138.0, 130.2, 129.1, 128.7, 127.1, 126.7, 116.1, 115.3, 65.3, 35.(KIM et al., 2010).


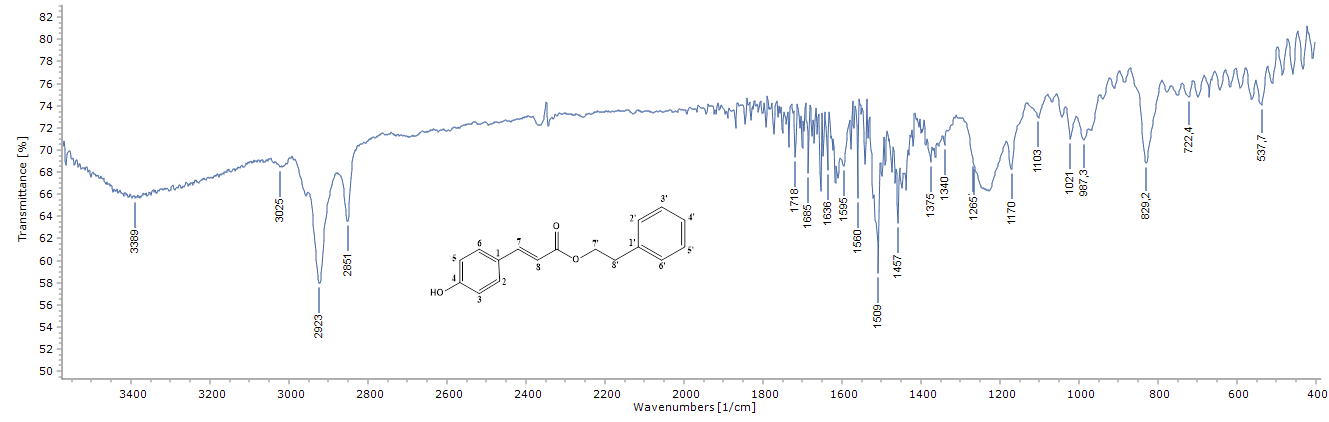


**Figure 52:** Infrared spectrum ʋmax (KBr, cm^-1^) of compound **17**

**Figure S53:** ^1^H NMR spectrum (400 MHz, CDCl_3_) of compound **17**

**Figure54:** ^13^C NMR spectrum (APT, 100 MHz, CDCl_3_) of compound **17**

**Figure S55:** ^13^C NMR spectrum (APT, 100 MHz, CDCl_3_) of compound **17**

**(*E*)-Naphthalene *p*-coumarate** (**18**):

**
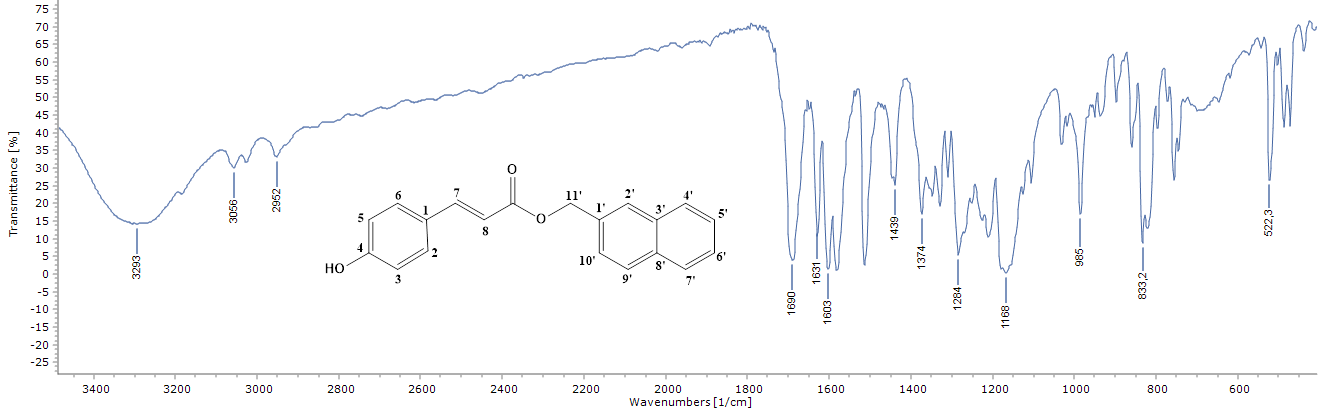
**

**Figure S56:** Infrared spectrum ʋmax (KBr, cm^-1^) of compound **18**

**Figure S57:** ^1^H NMR spectrum (400 MHz, DMSO-d_6_) of compound **18**

**Figure S58:** ^13^C NMR spectrum (APT, 100 MHz, DMSO-d_6_) of compound **18**


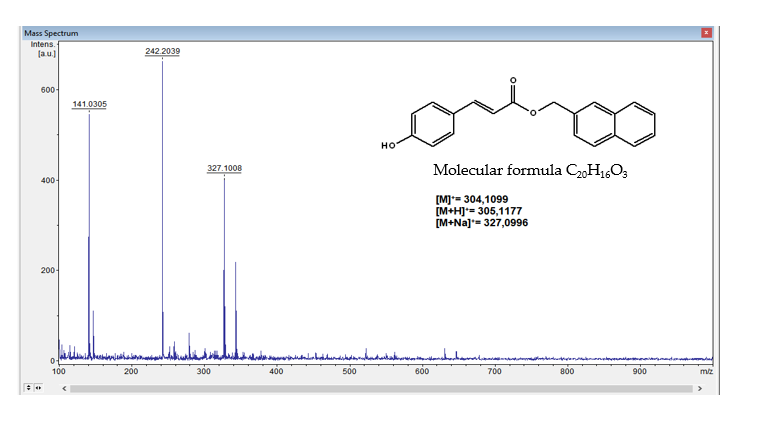


**Figure** S59: HRMS spectrum of compound **18**

**(*E*)-Hexyl 3(-4 propoxyphenyl)acrylate** (**19**)**:**


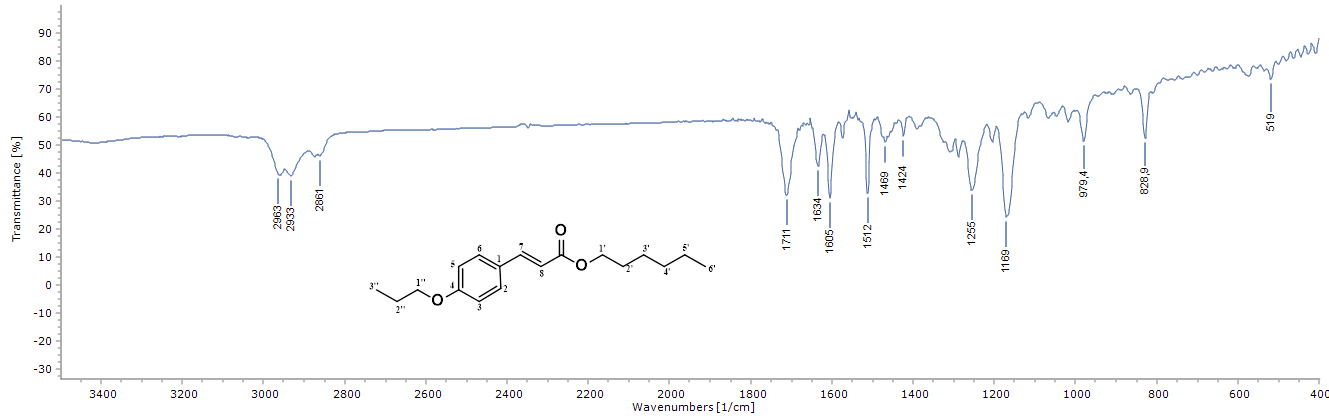


**Figure S60:** Infrared spectrum ʋmax (KBr, cm^-1^) of compound **19**

**Figure S61:** ^1^H NMR spectrum (500 MHz, CDCl_3_) of compound **19**

**Figure S62**: ^13^C NMR spectrum (APT, 100 MHz, CDCl_3_) of compound **19**


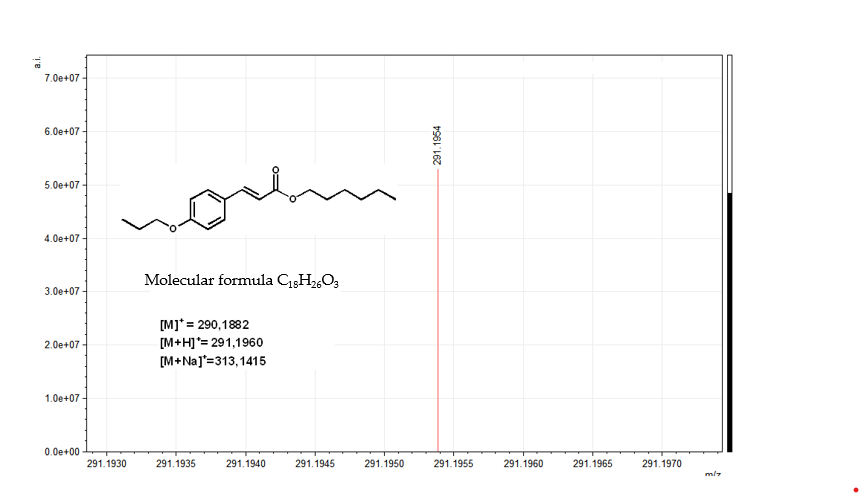


**Figura S63:** HRMS spectrum of compound **19**

**(E)-Hexyl 3-(4-Acetoxyphenyl)acrylate** (**20**)**:**


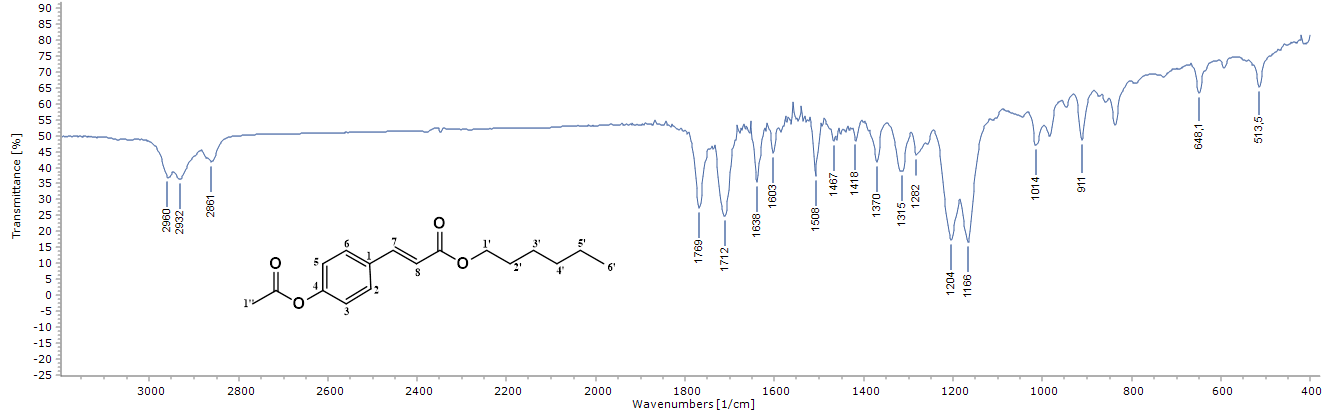


**Figure S64:** Infrared sprectrum ʋmax (KBr, cm^-1^) of compound **20**

**Figure S65:** ^1^H NMR spectrum (400 MHz, CDCl_3_) of compound **20**

**Figure S66:** ^13^C NMR spectrum (APT, 100 MHz, CDCl_3_) of compound **20**

**
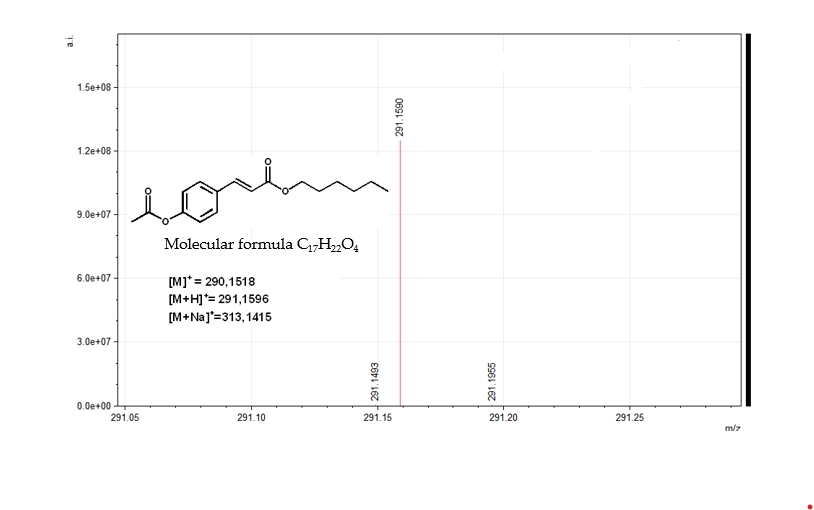
**

**Figure S67:** HRMS spectrum of compound **20**

**(*E*)-Hexyl 3-(4-Biphenylyl)acrylate** (**21**)**:**


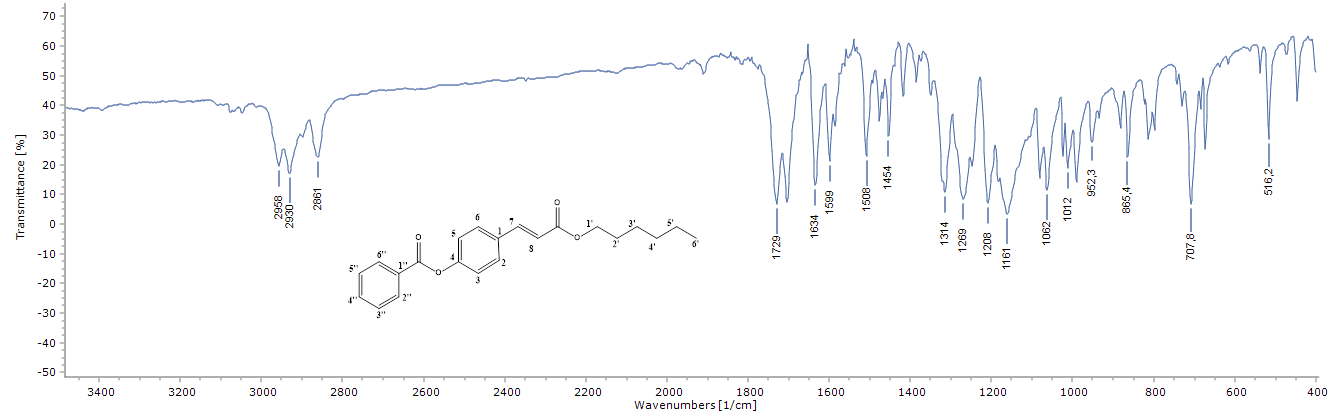


**Figure S68:** Infrared spectrum ʋmax (KBr, cm^-1^) of compound **21**

**Figure S69S:** ^1^H NMR spectrum (400 MHz, CDCl_3_) of compound **21**

**Figure S70:** ^13^C NMR spectrum (APT, 100 MHz, CDCl_3_) of compound **21**

**Figure S71:** ^13^C NMR spectrum (APT, 100 MHz, CDCl_3_) of compound **21**

**
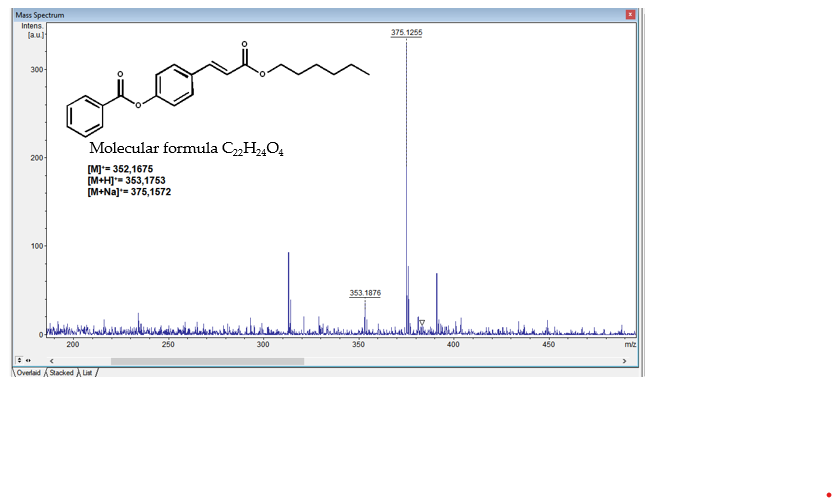
**

**Figure 72:** HRMS spectrum of compound **21**

**(*E*)-Hexyl 3-bromobenzyl-3-(4-phenylyl)acrylate** (**23**)**:**


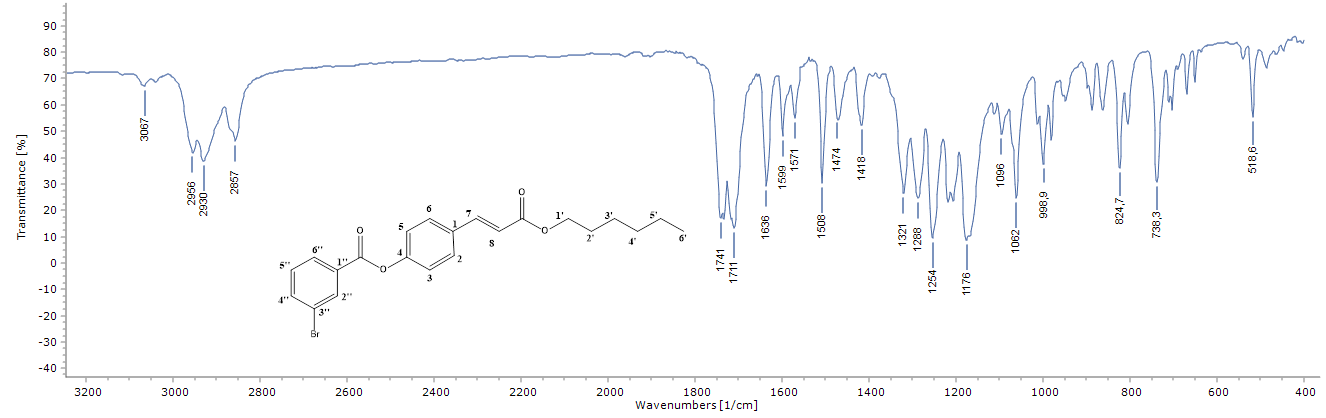


**Figure S73:** Infrared sprectrum ʋmax (KBr, cm^-1^) of compound **21**

**Figure S74:** ^1^H NMR spectrum (400 MHz, CDCl_3_) of compound **22**

**Figure S75:** ^13^C NMR spectrum (APT, 100 MHz, CDCl_3_) of compound **22**

**Figure S76:** ^13^C NMR spectrum (APT, 100 MHz, CDCl_3_) of compound **22**

**
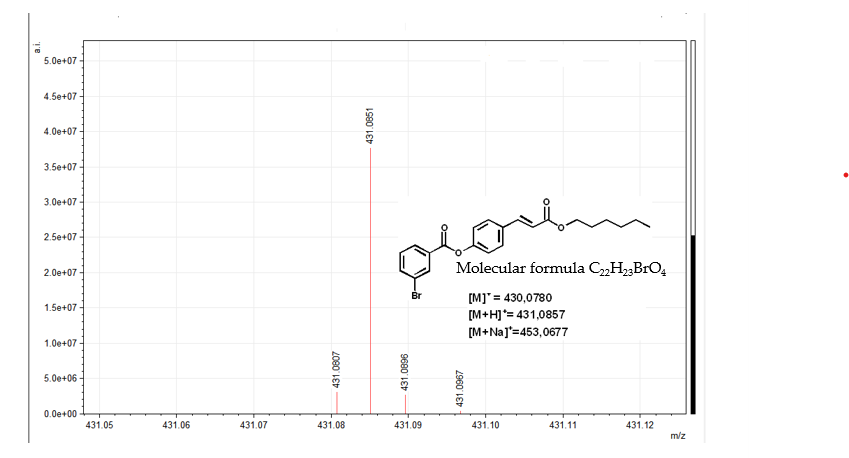
**

**Figure S77:** HRMS spectrum of compound **22**

**References**

1. Allegretta, G.; Weidel E.; Empting M.; Hartmann R. W. Catechol-based substrates of chalcone synthase as a scaffold for novel inhibitors of PqsD. *Eur J Med Chem*. **2015**, 90, 351–359.
2. Khatkar, A.; Nanda, A.; Kumar, P.; Narasimhan, B. Synthesis, antimicrobial evaluation and QSAR studies of *p*-coumaric acid derivaties. *Arab J Chem*. **2017**, 10, 3804-3815.
3. Hixson, J. L.; Sleep, N. R.; Capone, D. L.; Elsey, G. M.; Curtin, C. D.; Sefton, M. A.; Taylor, D. K. Hydroxycinnamic Acid Ethyl Esters as Precursors to Ethylphenols in Wine. *J.* *Agric. Food Chem*. **2012**, 60(9), 2293–2298.
4. Hosseini, R.; Moosavi, F.; Rajaian, H.; Silva, T.; Silva, D. M.; Soares, P.; Saso, L.; Edraki, N.; Miri, R.; Borges, F.; Firuzi, O. Discovery of neurotrophic agents based on hydroxycinnamic acid scaffold. *Chem Biol Drug Des*. **2016**, 88(6), 926–937.
5. Khatkar, A.; Nanda, A.; Kumar, P.; Narasimhan, B. Synthesis, antimicrobial evaluation and QSAR studies of *p*-coumaric acid derivaties. *Arab J Chem*. **2017**, 10, 3804-3815.
6. Nishimura, K.; Takenaka, Y.; Kishi, M.; Tanahashi, T.; Hiromi Yoshida, H.; Okuda, C.; Mizushin, Y. Synthesis and DNA Polymerase α and β Inhibitory Activity of Alkyl *p*-Coumarates and Related Compounds. *Chem Pharm Bull*. **2009**, 57(5), 476- 480.
7. Kim, K. D.; Min, H. S.; Yum, E. K.; Ok, O. S.; Ju, Y. W.; Chang, M. S. Melanogenesis Inhibition by Mono-hydroxycinnamic Ester Derivatives in B16 Melanoma Cells. *Bull Korean Chem Soc*. **2010**, 31(1), 181-184.
8. Lopes, S.P.; Castillo, Y.P.; Monteiro, M.L.; Menezes, R.R.P.P.B.; Almeida, R.N.; Martins, A.M.C.; Sousa, D.P. Trypanocidal Mechanism of Action and in silico Studies of *p*-Coumaric Acid Derivatives. Int. J. Mol. Sci. 2019, 20, 5916.
9. Lopes, S.P.; Yepes, L.M.; Pérez-Castillo, Y.; Robledo, S.M.; de Sousa, D.P. Alkyl and Aryl Derivatives Based on *p*-Coumaric Acid Modification and Inhibitory Action against *Leishmania braziliensis* and *Plasmodium falciparum*. Molecules **2020**, 25, 3178.
